# Biology of the sauropod dinosaurs: the evolution of gigantism

**DOI:** 10.1111/j.1469-185X.2010.00137.x

**Published:** 2011-02

**Authors:** P Martin Sander, Andreas Christian, Marcus Clauss, Regina Fechner, Carole T Gee, Eva-Maria Griebeler, Hanns-Christian Gunga, Jürgen Hummel, Heinrich Mallison, Steven F Perry, Holger Preuschoft, Oliver W M Rauhut, Kristian Remes, Thomas Tütken, Oliver Wings, Ulrich Witzel

**Affiliations:** 1 Steinmann Institute, Division of Palaeontology, University of BonnNussallee 8, 53115 Bonn, Germany; 2 Institut für Biologie und Sachunterricht und ihre Didaktik, University of FlensburgAuf dem Campus 1, 24943 Flensburg, Germany; 3 Clinic for Zoo Animals, Exotic Pets and Wildlife, University of ZurichWinterthurerstr. 260, 8057 Zurich, Switzerland; 4 Bayerische Staatssammlung für Paläontologie und Geologie, University of MunichRichard-Wagner-Strasse 10, 80333 Munich, Germany; 5 Institut für Zoologie, Abteilung Ökologie, University of MainzJohann-Joachim-Becher Weg 13, 55128 Mainz, Germany; 6 Zentrum für Weltraummedizin Berlin, Institut für Physiologie, Charite-University of BerlinArnimallee 22, 14195 Berlin, Germany; 7 Institut für Tierwissenschaften, University of BonnEndenicher Allee 15, 53115 Bonn, Germany; 8 Museum für Naturkunde, Leibniz-Institut für Evolutions- und Biodiversitätsforschung an der Humboldt-Universität zu BerlinInvalidenstrasse 43, 10115 Berlin, Germany; 9 Institut für Zoologie, Morphologie und Systematik, University of BonnPoppelsdorfer Schloss, 53115 Bonn, Germany; 10 Institut für Anatomie, Abteilung für Funktionelle Morphologie, University of BochumUniversitätsstrasse 150, 44801 Bochum, Germany; 11 Steinmann Institute, Division of Mineralogy, University of BonnPoppelsdorfer Schloss, 53115 Bonn, Germany; 12 Institut für Konstruktionstechnik, Fakultät für Maschinenbau, University of BochumUniversitätsstrasse 150, 44801 Bochum, Germany

**Keywords:** Dinosauria, Sauropoda, gigantism, Mesozoic, long neck, phylogenetic heritage, evolutionary innovation

## Abstract

The herbivorous sauropod dinosaurs of the Jurassic and Cretaceous periods were the largest terrestrial animals ever, surpassing the largest herbivorous mammals by an order of magnitude in body mass. Several evolutionary lineages among Sauropoda produced giants with body masses in excess of 50 metric tonnes by conservative estimates. With body mass increase driven by the selective advantages of large body size, animal lineages will increase in body size until they reach the limit determined by the interplay of bauplan, biology, and resource availability. There is no evidence, however, that resource availability and global physicochemical parameters were different enough in the Mesozoic to have led to sauropod gigantism.

We review the biology of sauropod dinosaurs in detail and posit that sauropod gigantism was made possible by a specific combination of plesiomorphic characters (phylogenetic heritage) and evolutionary innovations at different levels which triggered a remarkable evolutionary cascade. Of these key innovations, the most important probably was the very long neck, the most conspicuous feature of the sauropod bauplan. Compared to other herbivores, the long neck allowed more efficient food uptake than in other large herbivores by covering a much larger feeding envelope and making food accessible that was out of the reach of other herbivores. Sauropods thus must have been able to take up more energy from their environment than other herbivores.

The long neck, in turn, could only evolve because of the small head and the extensive pneumatization of the sauropod axial skeleton, lightening the neck. The small head was possible because food was ingested without mastication. Both mastication and a gastric mill would have limited food uptake rate. Scaling relationships between gastrointestinal tract size and basal metabolic rate (BMR) suggest that sauropods compensated for the lack of particle reduction with long retention times, even at high uptake rates.

The extensive pneumatization of the axial skeleton resulted from the evolution of an avian-style respiratory system, presumably at the base of Saurischia. An avian-style respiratory system would also have lowered the cost of breathing, reduced specific gravity, and may have been important in removing excess body heat. Another crucial innovation inherited from basal dinosaurs was a high BMR. This is required for fueling the high growth rate necessary for a multi-tonne animal to survive to reproductive maturity.

The retention of the plesiomorphic oviparous mode of reproduction appears to have been critical as well, allowing much faster population recovery than in megaherbivore mammals. Sauropods produced numerous but small offspring each season while land mammals show a negative correlation of reproductive output to body size. This permitted lower population densities in sauropods than in megaherbivore mammals but larger individuals.

Our work on sauropod dinosaurs thus informs us about evolutionary limits to body size in other groups of herbivorous terrestrial tetrapods. Ectothermic reptiles are strongly limited by their low BMR, remaining small. Mammals are limited by their extensive mastication and their vivipary, while ornithsichian dinosaurs were only limited by their extensive mastication, having greater average body sizes than mammals.

## CONTENTS

- Introduction 119
  1. General introduction 119
  2. Importance of body size 120
  3. Methods of estimating body mass in dinosaurs 120
  4. Unique body size of sauropods and theropods 123
  5. Cope's Rule in Sauropodomorpha and selective advantages of large body size 123
  6. Diversity of the Sauropoda 125
  7. Sauropodomorph phylogeny and evolution 125
- Bauplan and biology of sauropod dinosaurs 126
  1. Bauplan and skeletal anatomy 127
  2. Musculature reconstruction and locomotor evolution 128
  3. Locomotion: gait and speed 128
  4. Integument 129
  5. Respiratory system 129
  6. Dentition and digestive system 129
  7. Circulatory system 130
  8. Nervous system and sense organs 130
  9. Organ size and its scaling 131
  10. Physiology and thermoregulation 131
    - Lines of evidence 131
    - Bonehistologic evidence 132
    - Scaling effects: gigantothermy and ontogenetic change 132
    - Synthesis 133
  11. Life history, growth, and reproduction 133
- Body size evolution in sauropodomorpha 134
  1. Body size in basal dinosauriforms and basal sauropodomorphs 134
  2. Body size in early and basal sauropods 134
  3. Body size in Neosauropoda 135
  4. Independent gigantism in several lineages 135
  5. Island dwarfing 136
  6. Body size evolution and Cope's Rule 136
- Hypotheses explaining giant body size 136
  1. Limits to body size 136
  2. Resource availability 137
- More resources available through different boundary conditions 137
  1. Physical boundary conditions 137
    - Increased oxygen content of atmosphere 137
    - Increased plant productivity through increased CO2 content of the atmosphere 138
    - Higher ambient temperatures 139
  2. Biological boundary conditions 140
    - More nutritious food 140
    - Exceptionally productive habitats: mangroves and tidal flats 140
- More resources available through evolutionary innovation 140
  1. Long neck 140
    - First hypothesis: extension of reach 141
    - Second hypothesis: large feeding envelope versus acceleration of whole body 141
  2. Feeding 141
  3. Greater digestive efficiency 142
  4. Avian-style respiratory system 142
- Fewer resources used 143
  1. Reduction in body density 143
    - Superior skeletal materials 143
    - Light-weight construction 143
  2. Reduced cost of locomotion 144
  3. Reduced cost of respiration 144
  4. Lower basal metabolic rate and gigantothermy 144
  5. Reduced cost of reproduction 144
- Faster population recovery and faster individual growth 145
  1. Ovipary and gigantism 145
  2. Survivorship, high growth rate, and high BMR 145
- Historical contingency 145
  1. Decreased oxygen content of atmosphere 145
  2. Poor food quality 145
- Discussion 146
- Conclusions 148
- Acknowledgments 148
- References 148

## I. INTRODUCTION

### (1) General introduction

**Fig. 1:**
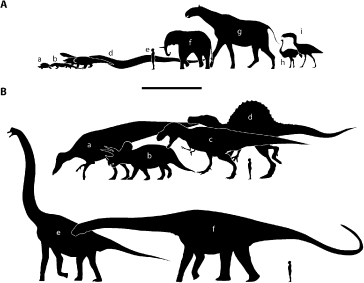
The largest representatives of different terrestrial vertebrate clades, both extant and extinct. (A) Non-dinosaurian terrestrial vertebrates and birds: (a) the tortoise *Geochelone gigantea*, (b) the Komodo dragon *Varanus komodoensis*, (c) the Pleistocene Australian monitor † *Varanus* (*Megalania*) *prisca*, (d) the Eocene boid snake † *Titanoboa cerrejonensis*, (e) *Homo sapiens*, (f) the African elephant *Loxodonta africana*, (g) the long-necked Oligocene rhinoceros † *Paraceratherium* (*Indricotherium*) *transouralicum*, (h) *Struthio camelus*, (i) an unnamed Miocene † Phorusracidae. (B) non-avian dinosaurs: (a) the hadrodaur † *Shantungosaurus giganteus*, (b) the ceratopsian † *Triceratops horridus*, (c) the theropod †*Tyrannosaurus rex*, (d) the theropod † *Spinosaurus aegyptiacus*, (e) the sauropod † *Brachiosaurus brancai*, (f) the sauropod †*Argentinosaurus huinculensis*. Scale = 5 m.

Body size is one of the most fundamental attributes of any organism (Hunt & Roy, 2005; Bonner, 2006). While some body size maxima (and minima) can be observed and studied directly in living organisms (e.g. the largest trees and the largest marine vertebrates), others have occurred in the geologic past. These must be studied from the fossil record, e.g. the largest insects (giant dragonflies of the Carboniferous), the largest terrestrial predators (theropod dinosaurs), and the largest terrestrial animals ever, the sauropod dinosaurs (Fig. 1). Their uniquely gigantic body size commands special interest from an evolutionary perspective. Sauropod dinosaurs represent a hugely successful radiation of herbivores that originated in the Late Triassic, dominated terrestrial ecosystems in the Jurassic, and flourished until the very end of the Cretaceous (Curry Rogers & Wilson, 2005; Tidwell & Carpenter, 2005). The aim of this paper is to review the evolution of gigantism in sauropod dinosaurs and to discuss and explore hypotheses explaining their unique body size.

Body size may either be expressed as linear dimensions, such as total length or height, or as body mass. Body mass is more relevant to most biological processes and thus is most commonly used throughout this review. Since sauropod skeletons are often incompletely preserved and the femur is the largest bone in the sauropod skeleton, its length is a good proxy for body size (Carrano, 2006), be it defined as linear dimensions or as body mass.

**Fig. 2:**
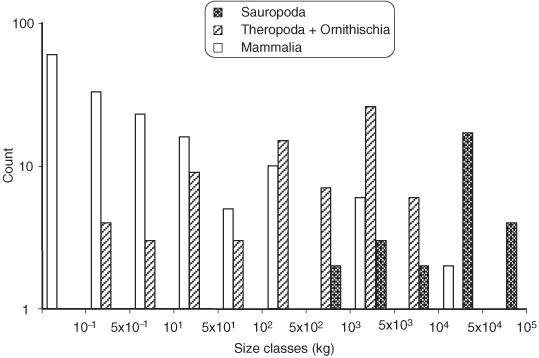
Comparison of body masses of sauropod dinosaurs, theropod and ornithischian dinosaurs and mammals. The mass data for sauropods are found in Table 1, while those for the other dinosaurs are primarily from Seebacher (2001) with additional data from Christiansen (1997) and Anderson *et al.* (1985). The data for mammals were compiled from Janis & Carrano (1992), Fortelius & Kappelman (1993), and Spoor *et al.* (2007). With the exception of the two largest forms they represent extant mammals only. Mammals show a strongly right-skewed distribution, theropods and ornithischians show intermediate masses, and sauropods show a strongly left-skewed distribution. Not that the *y*-axis is logarithmic.

Large body size evolved very early on and remained a hallmark throughout sauropod evolution (Dodson, 1990). The discrepancy in body size between other dinosaurs and sauropods, as well as between the largest land mammals and sauropods (Figs 1, 2), has recently been highlighted by the availability of more accurate mass estimates (see Table 1) calculated from volume estimates based on photogrammetric measurements of actual skeletons (Gunga *et al.*, 2007, 2008; Stoinski, Suthau & Gunga, in press) or based on scientific reconstructions (e.g. Paul, 1987, 1997a; Henderson, 1999, 2006; Seebacher, 2001). These estimates place common sauropods consistently in the 15–40 t category (Table 1). In addition, there are a number of very large sauropods, e.g. the basal macronarian *Sauroposeidon* (Wedel, Cifelli & Sanders, 2000*a*, *b*) and the titanosaur *Argentinosaurus*, for which published estimates (reviewed in Mazzetta, Christiansen & Farina, 2004) are a staggering 70–90 t! Small sauropod species with an adult body mass of less than 4–5 t are almost unknown (Table 1) with the exception of several dwarf forms from palaeo-islands (Weishampel, Grigorescu & Norman, 1991; Jianu & Weishampel, 1999; Dalla Vecchia, 2005; Sander *et al.*, 2006; Benton *et al.*, 2010; Stein *et al.*, in press).

**Table 1:** Compilation of body mass estimates for selected sauropods from the literature. The table lists those species for which reliable estimates are available because of abundant and complete fossil material and the largest valid sauropod species (in bold) which are known from less complete material. It also intends to show the variation of estimates obtained by different methods

Taxon	Reference	Mass (kg)	Method of mass estimate
*Amargasaurus cazaui*	Seebacher (2001)	6853	polynomial volume
***Amphicoelias fragillimus***	Paul (1998)	**90000–150000**	method not given
*Anchisaurus sinensis*	Seebacher (2001)	84	polynomial volume
***Antarctosaurus giganteus***	Mazzetta *et al.* (2004)	**69000**	regression analysis
*Antarctosaurus wichmannianus*	Mazzetta *et al.* (2004)	33410	regression analysis
*Antarctosaurus wichmannianus*	Mazzetta *et al.* (2004)	24617	regression analysis
*Apatosaurus louisae*	Colbert (1962)	32420	scale model
*Apatosaurus louisae*	Anderson *et al.* (1985)	30000–37500	long bone circumference
*Apatosaurus louisae*	Alexander (1989)	34000–35000	scale model
*Apatosaurus louisae*	Christiansen (1997)	19500	scale model
*Apatosaurus louisae*	Paul (1998)	17500	method not given
*Apatosaurus louisae*	Seebacher (2001)	22407	polynomial volume
*Apatosaurus louisae*	Foster (2007)	34035	long bone circumference
*Apatosaurus louisae* (juvenile)	Foster (2007)	4254	long bone circumference
*Apatosaurus louisae*	Packard *et al.* (2009)	18000	nonlinear regression analysis
*Apatosaurus* sp.	Erickson *et al.* (2001)	25952	long bone circumference
***Argentinosaurus huinculensis***	Mazzetta *et al.* (2004)	**72936**	regression analysis
*Barosaurus lentus*	Foster (2007)	11957	long bone circumference
*Barosaurus* sp.	Seebacher (2001)	20040	polynomial volume
*Brachiosaurus altithorax*	Paul (1998)	35000	method not given
*Brachiosaurus altithorax*	Seebacher (2001)	28265	polynomial volume
*Brachiosaurus altithorax*	Foster (2007)	43896	long bone circumference
*Brachiosaurus brancai*	Janensch (1938)	40000	method not given
*Brachiosaurus brancai*	Colbert (1962)	78260	scale model
*Brachiosaurus brancai*	Anderson *et al.* (1985)	31600	long bone circumference
*Brachiosaurus brancai*	Anderson *et al.* (1985)	29335	long bone circumference
*Brachiosaurus brancai*	Alexander (1985)	46600	scale model
*Brachiosaurus brancai*	Paul (1988)	45000–50000	method not given
*Brachiosaurus brancai*	Alexander (1989)	47000	scale model
*Brachiosaurus brancai*	Christiansen (1997)	37400	scale model
*Brachiosaurus brancai*	Gunga *et al.* (1999)	74420	stereophotogrammetry and laser scanning of mounted skeleton
*Brachiosaurus brancai*	Mazzetta *et al.* (2004)	39500	scale model
*Brachiosaurus brancai*	Gunga *et al.* (2008)	38000	laser scanning of mounted skeleton
*Brachiosaurus brancai*	Packard *et al.* (2009)	16000	nonlinear regression analysis
*Camarasaurus grandis*	Foster (2007)	18413	long bone circumference
*Camarasaurus grandis*	Foster (2007)	9321	long bone circumference
*Camarasaurus lewisi*	Seebacher (2001)	11652	polynomial volume
*Camarasaurus supremus*	Christiansen (1997)	8800	scale model
*Camarasaurus supremus*	Mazzetta *et al.* (2004)	9300	scale model
*Cetiosaurus oxoniensis*	Mazzetta *et al.* (2004)	15900	scale model
*Dicraeosaurus hansemanni*	Christiansen (1997)	5400	scale model
*Dicraeosaurus hansemanni*	Gunga *et al.* (1999)	12800	stereophotogrammetry and laser scanning of mounted skeleton
*Dicraeosaurus hansemanni*	Mazzetta *et al.* (2004)	5700	scale model
*Diplodocus carnegii*	Christiansen (1997)	15200	scale model
*Diplodocus carnegii*	Foster (2007)	12657	long bone circumference
*Diplodocus carnegii*	Foster (2007)	12000	long bone circumference
*Diplodocus* sp.	Colbert (1962)	10560	scale model
*Diplodocus* sp.	Anderson *et al.* (1985)	5000–15000	long bone circumference
*Diplodocus* sp.	Henderson (1999)	13421	3-D mathematical slicing
*Diplodocus* sp.	Packard *et al.* (2009)	4000	nonlinear regression analysis
*Euhelopus zdanskyi*	Paul (1997*b*)	3800	scale model
*Europasaurus holgeri*	Stein, unpublished data	690	long bone circumference
*Haplocanthosaurus delfsi*	Foster (2007)	21000	long bone circumference
*Haplocanthosaurus priscus*	Foster (2007)	10500	long bone circumference
*Haplocanthosaurus* sp.	Paul (1997*b*)	12800	scale model
*Haplocanthosaurus* sp.	Seebacher (2001)	14529	polynomial volume
*Janenschia* sp.	Lehman & Woodward (2008)	14029	long bone circumference
*Lufengosaurus huenei*	Seebacher (2001)	1193	polynomial volume
*Magyarosaurus dacus*	Stein, unpublished data	900	long bone circumference
*Mamenchisaurus hochuanensis*	Christiansen (1997)	14300	scale model
*Mamenchisaurus hochuanensis*	Seebacher (2001)	18170	polynomial volume
*Mamenchisaurus hochuanensis*	Mazzetta *et al.* (2004)	15100	scale model
*Massospondylus* sp.	Seebacher (2001)	137	polynomial volume
*Omeisaurus tianfunensis*	Christiansen (1997)	9800	scale model
*Omeisaurus tianfunensis*	Seebacher (2001)	11796	polynomial volume
*Omeisaurus tianfunensis*	Mazzetta *et al.* (2004)	9800	scale model
*Opisthocoelicaudia skarzynskii*	Anderson *et al.* (1985)	22000	long bone circumference
*Opisthocoelicaudia skarzynskii*	Paul (1997*b*)	8400	scale model
*Opisthocoelicaudia skarzynskii*	Seebacher (2001)	10522	polynomial volume
*Opisthocoelicaudia skarzynskii*	Packard *et al.* (2009)	13000	nonlinear regression analysis
***Paralititan stromeri***	Burness *et al.* (2001)	**59000**	method not given
*Patagosaurus* sp.	Seebacher (2001)	9435	polynomial volume
*Plateosaurus engelhardti*	Seebacher (2001)	1073	polynomial volume
*Plateosaurus engelhardti*	Gunga *et al.* (2007)	630–912	laser scanning of mounted skeleton
*Riojasaurus* sp.	Seebacher (2001)	3039	polynomial volume
***Sauroposeidon proteles***	Wedel *et al.* (2000*a*)	**50000–60000**	method not given
***Seismosaurus halli***	Gillette, (1994)	**100000**	method not given
*Seismosaurus halli*	Seebacher (2001)	49276	polynomial volume
*Seismosaurus hallorum*	Foster (2007)	42500	method not given
*Shunosaurus lii*	Christiansen (1997)	3400	scale model
*Shunosaurus lii*	Seebacher (2001)	4793	polynomial volume
*Shunosaurus lii*	Mazzetta *et al.* (2004)	3600	scale model
***Supersaurus vivianae***	Foster (2007)	**40200**	method not given
*Thecodontosaurus antiquus*	Seebacher (2001)	25	polynomial volume
***Ultrasauros macintoshi***	Paul (1998)	**45000–50000**	method not given

The largest representatives of all other dinosaur lineages, despite being very big in general perception, rarely exceeded the 10 t threshold and thus actually are in the size range of very large terrestrial mammals such as the fossil indricotheres (Fortelius & Kappelman, 1993) and extant and fossil elephants (Fig. 2). Among animals, only whales grow to a body mass larger than sauropods, but a direct comparison between these two groups is not very meaningful because of the fundamentally different constraints of the aquatic *versus* the terrestrial environment.

### (2) Importance of body size

Body size is fundamentally linked to the bauplan, life history, and ecology of any organism (Clutton-Brock & Harvey, 1983; Peters, 1983; Schmidt-Nielsen, 1984; Alexander, 1998; Hunt & Roy, 2005; Makarieva, Gorshkov & Li, 2005; Bonner, 2006), each bauplan having its lower and upper limit at which it can function. In addition, body size evolution and implications of body size for other species characteristics have received an increasing amount of attention in recent years because it has been realized that evolutionary innovation is closely tied to body size changes in evolutionary lineages. Miniaturization may lead to new designs, and body size decrease and increase is coupled with heterochrony leading to changes in morphology (Long & McNamara, 1995, 1997*a*, *b*; McNamara, 1997; McNamara & McKinney, 2005).

### (3) Methods of estimating body mass in dinosaurs

Any discussion of gigantism in sauropod dinosaurs requires reliable estimates of their body mass. Highly disparate estimates can be found in the literature (Peczkis, 1994; see also Table 1), mainly due to different methods employed. Mass estimates are generally either based on some measure of volume that is then converted into body mass or on a biomechanical approach, e.g. using long bone circumference (Anderson, Hall-Martin & Russell, 1985; corrected by Alexander, 1989; see also Packard, Boardman & Birchard, 2009; Cawley & Janacek, 2010). Each method has different sources of error, and the main advantages and disadvantages of some of these methods have been intensively discussed in the literature (Colbert, 1962; Lambert, 1980; Schmidt-Nielsen, 1984, 1997; Anderson *et al.*, 1985; Haubold, 1990; Gunga *et al.*, 1999; Paul, 1997*b*, Henderson, 1999; Seebacher, 2001; Motani, 2001; Christiansen & Fariña, 2004; Mazzetta *et al.*, 2004; Foster, 2007; Packard *et al.*, 2009).

One method for estimating body mass based on reconstructed body volume involves three-dimensional photogrammetry of actual skeletons using a laser scanner (Gunga *et al.*, 1999; 2007, 2008; Bates *et al.*, 2009; Stoinski *et al.*, in press). Advantages of this approach include that geometrical calculations can be made easily based on the respective body parts, and that different hypothetical body shapes, resulting in different body masses, can be tested (Gunga *et al.*, 2007, 2008; Bates *et al.*, 2009; Stoinski *et al.*, in press). Segment masses can also easily be obtained. Finally, with photogrammetrical methods, measurement errors are also partitioned and do not affect the entire estimate. In mass estimated based on long bone circumference, on the other hand, whenever a local measurement error occurs (e.g. due to deformation during fossilization), the direct result is that the total mass of the animal is calculated incorrectly. A similar method is based on creating 3D skeletal mounts from digitized bones, and using these instead of laser-scanned mounts (Mallison, 2007, in press *b*). This allows easy correction of errors in mounts and thus revisions.

Recent work by Wedel (2005) suggests that volume-based estimates are generally too high because they are based on a specific density in a living sauropod of 0.9–1 kg L^−1^, as in modern crocodilians. However, it is becoming generally accepted that because of their extensively pneumatized axial skeleton (Perry, 2001; Henderson, 2004; Wedel, 2003*a*, *b*, 2005, 2009; Schwarz & Fritsch, 2006) living sauropods probably had a specific density of about 0.8 kg L^−1^ (Wedel, 2005), which is more like that of birds (0.73 kg L^−1^, Hazlehurst & Rayner, 1992). Wedel (2005) accordingly suggested that volume-based mass estimates published before the modern consensus on pneumatized skeletons should be reduced by about 10%.

A caveat to the tissue density of 0.8 kg L^−1^ given by Wedel (2005), and a novel method for estimating body mass, is offered by a recent allometric study on the dimensions of semicircular canals (SCC) in the skull (Clarke, 2005). Plotting SCC diameter of the Berlin specimen of *Brachiosaurus* (recently assigned to a new genus, *Giraffatitan*, based on numerous differences from the type species, *B. altithorax*; Taylor, 2009) on a regression of SCC dimensions against body mass in extant amniotes, Clarke (2005) found that the dimensions of the posterior SCC are consistent with a body mass of about 75 t, while the anterior SCC suggests a higher mass and the lateral SCC a lower mass. At 30–50 t, the most recent volume-based estimates for this individual are considerably lower (Seebacher, 2001; Gunga *et al.*, 2008). A higher tissue density than 0.8 kg L^−1^ would result in higher body mass estimates and thus would be more consistent with the results of Clarke (2005).

### (4) Unique body size of sauropods and theropods

**Fig. 3:**
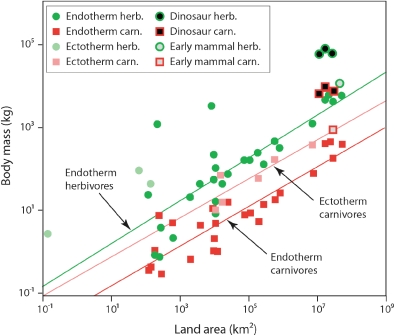
Body mass of the largest species inhabiting a land mass regressed against the size of the land mass in extant and Late Pleistocene terrestrial amniotes. The species are grouped by metabolism (bradymetabolic ectothermy *versus* tachymetabolic endothermy) and trophic level (herbivores *versus* carnivores). The two outliers of endothermic herbivores are island dwarf elephants. The largest species were ectothermic herbivores on only three land masses, precluding regression analysis of this group. Note that maximum body mass for a given land mass decreases with increasing metabolic rate and trophic level. Fossil mammal taxa adhere to the regressions while sauropod and theropod dinosaurs do not, being much larger than predicted. See text for details. Redrawn from Burness *et al.* (2001).

Dinosaurs have long been associated with extraordinary body size (Dodson, 1990), and estimates of maximal dinosaurian body size have received more than passing attention. Partially this is because of the innately human interest in identifying the largest ever representative of a group (Owen-Smith, 1988), which sometimes led to exaggerated claims of body mass for dinosaurs and fossil mammals (Fortelius & Kappelman, 1993). However, only recently has it been realized that two groups stand out among the dinosaurs from an ecological perspective, the Theropoda and the Sauropoda. While other studies (Janis & Carrano, 1992; Farlow, 1993; Paul, 1994, 1997*b*, 1998; Alexander, 1998) addressed this issue, that of Burness, Diamond & Flannery (2001) is most to the point. Regressing land mass size against body mass of the largest species inhabiting the land mass (top species) for recent and Pleistocene terrestrial tetrapods, Burness *et al.* (2001) observed that there is close correlation between these two variables when trophic level (herbivory *versus* carnivory) and metabolism (bradymetabolic ectothermy *versus* tachymetabolic endothermy) are taken into account (Fig. 3). The study included top species on land masses ranging from small oceanic islands of a few square kilometers in size to continents as large as Asia.

When adding the largest herbivorous and carnivorous dinosaurs then known to their dataset (Fig. 3), the estimated body masses of these species were an order of magnitude greater than predicted by the ectotherm regressions for the land mass they inhabited (Burness *et al.*, 2001). The largest herbivores in the study all belonged to Sauropoda (*Sauroposeidon*, *Argentinosaurus*, *Paralititan*) and the largest carnivores to Theropoda (*Tyrannosaurus*, *Giganotosaurus*, *Charcharodontosaurus*). Specifically, the theropods were an astounding 12 times heavier than predicted by the regression equations for extant ectotherms, and the difference for sauropods is also remarkable (1.5–3 times heavier than predicted). If both of these dinosaur groups were tachymetabolic endotherms, as we will argue below, the gap between prediction and observation is even larger. In fact, the magnitude of the gap led Burness *et al.* (2001) to suggest that dinosaurs must have been ectothermic. As predicted from energy loss between trophic levels (Burness *et al.*, 2001; Owen-Smith & Mills, 2008), the largest herbivores, the sauropods, are an order of magnitude larger than the largest carnivores (Fig. 3).

### (5) Cope's Rule in Sauropodomorpha and selective advantages of large body size

Sauropodomorpha, as an evolutionary lineage, started out with small animals of 10^1^ kg body mass (BM), such as *Saturnalia* (Langer *et al.*, 1999) and *Panphagia* (Martinez & Alcober, 2009) from the Carnian (early Late Triassic), from which the later giants with a BM of 10^5^ kg evolved. This profound evolutionary body size increase over four orders of magnitude begs the question of the applicability of Cope's Rule (Polly, 1998; Hone & Benton, 2005; Hone *et al.*, 2005; Carrano, 2006). In its most general formulation, Cope's Rule posits that body size tends to increase in evolutionary lineages over time, while more stringent versions either call for a general shift in average body size in a lineage from smaller to larger or for a general spread in the range of body sizes as evolutionary time progresses.

Bonner (2006) offered a rather simple (if not simplistic) explanation for Cope‘s Rule in its most general form, i.e. that as life diversifies, there is always room for body size to expand in one direction: to the top. As habitat is partitioned and ecospace becomes crowded, one way out is evolution towards larger body size (Bonner, 2006). However, this works only if body size in the specific evolving lineage has not yet met the physical limits of its bauplan and if the ecological carrying capacity allows for long-term survival of the population. Furthermore, Bonner‘s (2006) hypothesis only accounts for the increase in size range, but not for a general shift towards larger average body size—which is what happened in the sauropod lineage, in which even small taxa are one order of magnitude larger than their basal sauropodomorph ancestors.

Cope's Rule has been discussed controversially in the past, and its validity has not been universally accepted (Polly, 1998; Blankenhorn, 2000; Alroy, 1998, 2000; Knouft & Page, 2003; Moen, 2006). This discussion is not the focus of this review, but we obtain from it hypothesized selective advantages that drive body size evolution towards a larger average (Stanley, 1973; Clutton-Brock & Harvey, 1983; Blankenhorn, 2000; Hone & Benton, 2005; Table 2).

**Table 2:** Selective advantages and disadvantages of larger body size based on a compilation by Hone & Benton (2005)

Benefits of larger body size
-Increased defence against predation
-Increase in predation success
-Greater range of acceptable foods
-Increased success in mating
-Increased success in intraspecific competition
-Increased success in interspecific competition
-Extended longevity
-Increased intelligence (with increased brain size)
-At very large size, the potential for thermal inertia
-Survival through lean times and resistance to climatic variation and extremes
Problems caused by larger body size
-Increased vulnerability to predation
-Increased development time (both pre- and postnatal)
-Increased demand for resources
-Increased extinction risk because of:
-Longer generation time gives a slower rate of evolution, reducing the ability to adapt
-Lower abundance (i.e. small genetic pool, also reduces ability to adapt)
-Lower fecundity through reduced number of offspring

For herbivores the most important of these selective advantages (Table 2) is generally believed to be that larger body size decreases predation pressure. Strong evidence for this view was published most recently by Owen-Smith & Mills (2008). Two factors, the energy loss from one trophic level to the next and large size as predation protection, provide an explanation of why in modern terrestrial ecosystems the largest mammalian herbivores are an order of magnitude larger than the largest mammalian carnivores (Burness *et al.*, 2001). This is also the case in most dinosaur faunas in which the largest herbivore (generally a sauropod) is an order of magnitude larger than the largest predator, a theropod. Theropod body size thus may have been limited by sauropod body size. As sauropods reached a certain body size maximum, e.g. dictated by land mass size, so would theropods.

On the other hand, with predation pressure potentially being the dominant force driving evolutionary body size increase in herbivores, limitations to theropod body size other than prey availability (e.g. biomechanical limits to their bipedal body plan) may have affected maximum body size in sauropods. Once sauropods had evolved to a body size sufficient to protect them from theropod predation, their evolutionary size increase might have come to a halt because of the selective disadvantages of large body size (Table 2). Studies of African savannah ecosystems (Owen-Smith & Mills, 2008) suggest that the abundance of the largest herbivores, i.e. elephants, is limited by food abundance, not by predation pressure. Sauropods in the Late Jurassic Morrison Formation ecosystem are also hypothesized to have been food-limited. Through their capacity for outcompeting smaller animals in access to food and their relative immunity to predation, elephants may also limit the abundance of smaller herbivores and the trophic energy available for carnivores (Hummel & Clauss, 2008; Owen-Smith & Mills, 2008). If these observations were to apply to herbivory-based ecosystems in general, understanding sauropod biology and gigantism would hold the key to understanding Late Triassic to Cretaceous terrestrial ecosystems in general.

### (6) Diversity of the Sauropoda

Sauropod dinosaurs were a highly diverse group with over 90 valid genera known in 2005 (Upchurch & Barrett, 2005). New species are constantly being found (e.g. *Bonitasaura salgadoi*Apesteguía, 2004; *Brachytrachelopan mesai*Rauhut *et al.*, 2005; *Puertasaurus reuili*Novas *et al.*, 2005; *Turiasaurus riodevensis*Royo-Torres, Cobos & Alcalá, 2006; *Futalognkosaurus dukei*Calvo *et al.*, 2007; *Daxiatitan binglingi*You *et al.*, 2008; *Spinophorosaurus nigerensis*Remes *et al.*, 2009). The current tally is at 175 genera and approximately 200 species (Mannion & Upchurch, in press *a*, *b*), making the Sauropoda the most diverse of all major dinosaurian herbivore groups. They are also the longest-lived dinosaurian herbivore group, with the first sauropods being found in the Late Triassic (Yates & Kitching, 2003) and the last in the latest stages of the Maastrichtian (see Upchurch, Barrett & Dodson, 2004). Sauropods are known from all continents, including a first record from Antarctica (Smith & Pol, 2007). The recent finds reveal a remarkable diversity in body plans and feeding adaptations (Apesteguía, 2004; Rauhut *et al.*, 2005; Sereno *et al.*, 2007) which, together with the fragmentation of Pangea during the Jurassic and Cretaceous, may be responsible for the diversity increase through time.

Sauropods remain rare in the Lower Jurassic of China and South Africa, the only regions that have yielded an extensive terrestrial fossil record for this time interval. Until recently, it was believed that sauropod dinosaurs had their greatest diversity and ecological impact in the Late Jurassic and afterwards started to decline, becoming rare in the Late Cretaceous (Dodson, 1990; Weishampel & Norman, 1989). However, as dinosaur research entered the global age, it became apparent that this is a view centered on North America, and current discoveries suggest that many terrestrial ecosytems were dominated by sauropods to the very end of the Cretaceous.

### (7) Sauropodomorph phylogeny and evolution

**Fig. 4:**
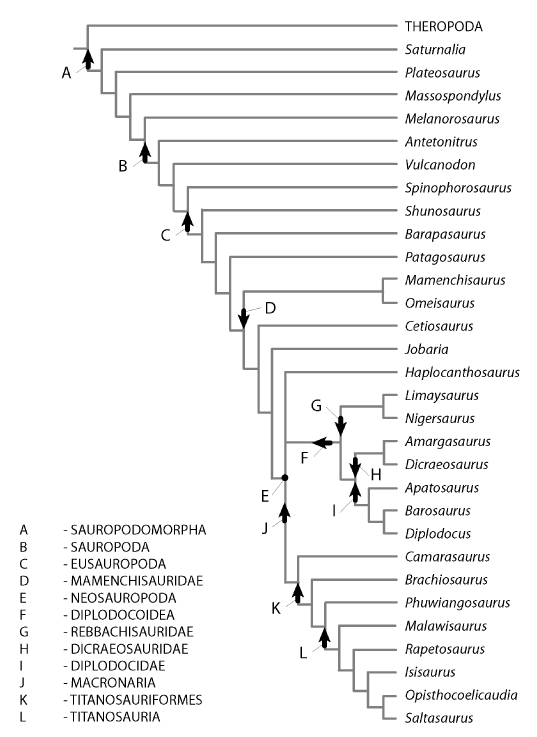
Simplified sauropod phylogeny compiled from Wilson (2002), Upchurch *et al.* (2007*a*), Yates (2007), Allain & Aquesbi (2008), and Remes *et al.* (2009). Only well-known taxa whose position in the phylogeny is relatively stable are shown. Arrows indicate stem-based taxa, and dots indicate node-based taxa.

The prerequisite for all enquiries into the evolution of body size, and gigantism in particular, are robust phylogenetic hypotheses (see Gould & MacFadden, 2004). These have only become available for sauropods in the last 15 years, through the work of J.A. Wilson (Wilson, 2002, 2005; Wilson & Upchurch, 2009; see also Wilson & Sereno, 1998), P. Upchurch (Upchurch *et al.*, 2004; see also Upchurch, 1995, 1998, 1999), and K. Curry Rogers on titanosaurs (Curry Rogers, 2005; see also Salgado, Coria & Calvo, 1997). These hypotheses largely agree on the general aspects of sauropod phylogeny (Fig. 4) with a consensus now having been reached (Wilson & Upchurch, 2009). Also, Taylor *et al.* (in press) define Sauropoda as all taxa closer to *Saltasaurus* than to *Melanorosaurus*, and hopefully this definition will lead to some systematic stability.

Recent discoveries (Buffetaut *et al.*, 2000, 2002) and phylogenetic work (Upchurch, Barrett & Galton, 2007*a*; Upchurch *et al.*, 2007*b*; Yates, 2007) reveal a number of taxa more basal than the traditionally recognized most basal sauropod *Vulcanadon* from the Lower Jurassic of Zimbabwe. However, the earliest evidence for a fully graviportal stance is only seen in *cf. Isanosaurus* from the Rhaetian of Thailand (Buffetaut *et al.*, 2002). Other basal taxa are *Kotasaurus* and *Barapasaurus* from the Lower Jurassic of India, *Shunosaurus* from the Middle Jurassic of China, and *Spinophorosaurus* from the Middle Jurassic of North Africa (Fig. 4). One particular clade of basal sauropods, the Mamenchisauridae (Fig. 4), seem to be an endemic, or near-endemic eastern Asian radiation (Russell, 1993; Upchurch, Hunn & Norman, 2002; Rauhut *et al.*, 2005; Wilson, 2005; Wilson & Upchurch, 2009) and include the sauropods with the relatively longest necks, such as *Omeisaurus* and *Mamenchisaurus*.

Advanced sauropods form a monophyletic clade called Neosauropoda (Upchurch, 1995, 1998; Wilson & Sereno, 1998; Wilson, 2002, 2005; Upchurch *et al.*, 2004; Harris, 2006), which is divided into two main lineages, the Diplodocoidea and the Macronaria (Fig. 4). Diplodocoids include the bizzarre rebbachisaurids (Lavocat, 1954; Calvo & Salgado, 1995; Sereno *et al.*, 1999, 2007) and dicraeosaurids (Janensch, 1914; Salgado & Bonaparte, 1991; Rauhut *et al.*, 2005) as well as the familiar diplodocids (Marsh 1884; Hatcher, 1901; Gilmore, 1936; Upchurch *et al.*, 2004) (Fig. 4). Whereas rebbachisaurids are so far only known from the Cretaceous, both dicraeosaurids and diplodocids appear in the Late Jurassic. Unequivocal records of diplodocids are not known from sediments younger than the latest Jurassic, whereas dicraeosaurids are still found in the Early and, possibly, the earliest Late Cretaceous (Stromer, 1932; Salgado & Bonaparte, 1991; Rauhut, 1999), and rebbachisaurids might have survived until the later stages of the Late Cretaceous (Sereno *et al.*, 2007). Macronarians are the most successful clade of sauropods (Fig. 4) and include the Late Jurassic *Camarasaurus* (e.g. Osborn & Mook, 1921), the Brachiosauridae (e.g. *Brachiosaurus* and *Cedarosaurus*), which flourished from the Late Jurassic to the Early Cretaceous (Riggs, 1903; Janensch, 1914; Wedel *et al.*, 2000*b*, Upchurch *et al.*, 2004) but may not be a natural grouping, and the titanosaurs, the most diverse and widespread clade of Cretaceous sauropods (Curry Rogers, 2005).

Titanosaurian anatomy is still poorly understood because most taxa are only known from a single or a few incomplete skeletons each and have not been studied in sufficient detail. Titanosaurs differ in several aspects of their locomotor apparatus from more basal sauropods, including their more widely spaced legs, documented by anatomical features and so-called ‘wide-gauge’ trackways (Wilson, 2005). A basal titanosaur known from abundant material is *Phuwiangosaurus* from the Lower Cretaceous of Thailand. Typical derived titanosaurs are *Rapetosaurus* from the latest Cretaceous of Madagascar (Fig. 4) and *Alamosaurus* from the latest Cretaceous of the southwestern USA.

## II. BAUPLAN AND BIOLOGY OF SAUROPOD DINOSAURS

We focus on those aspects of the sauropod bauplan and biology that are potentially informative on the gigantism issue. When describing the bauplan of a group like the sauropods, it is important to acknowledge that the consistency we observe in one organ system (e.g. the skeletal system with a generally ‘characteristic’ design) need not necessarily imply that other organ systems were of similar consistency across the species described. A good example for this, among the mammals, is provided by the primates which are a clearly defined group with a comparatively uniform skeletal bauplan, yet exhibiting an extreme variety of digestive tract designs, including both foregut- and hindgut fermentation (Chivers & Hladik, 1980).

### (1) Bauplan and skeletal anatomy

**Fig. 5:**
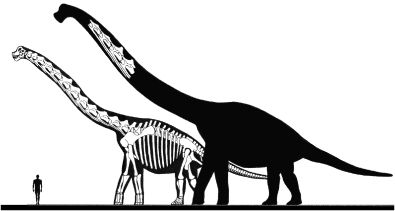
The sauropod body plan and body size. The reconstruction of *Brachiosaurus brancai* (recently renamed *Giraffatitan*, see Taylor, 2009) is based on the mounted skeleton in the Natural History Museum Berlin. *Sauroposeidon* from the Lower Cretaceous of Oklahoma (USA), one of the recently described truly gigantic sauropods, is only known from a string of four neck vertebrae. Based on these, the animal can be estimated to have been about 30% larger in linear dimension than the Berlin *Brachiosaurus*. Modified from Wedel *et al.* (2000*b*).

The sauropod body plan is unique among terrestrial tetrapods: a body superficially similar to that of proboscideans (elephants) among mammals is combined with a very small head on a very long neck and a tail, considerably exceeding those of other dinosaurs in relative (and absolute) length (Figs 1, 5). The small and light-weight skull is biomechanically linked to the long neck because of the leverage exerted by the head on the neck (Witzel & Preuschoft, 2005; Witzel, 2007; Witzel *et al.*, in press).

All sauropods were quadrupedal, graviportal animals with massive, columnar limbs supporting the body (Fig. 5). The fossil record suggests that the optimization of the forelimb towards a fully erect, parasagittally-swinging column was not an exaptation that allowed gigantism, but evolved in parallel (Remes, 2008), and that this parallel evolution was necessary for sauropods to attain a multi-tonne body mass.

With the exception of the basal macronarians *Brachiosaurus* (Fig. 5), and *Cedarosaurus*, the hindlimbs were considerably longer in sauropods than the forelimbs, and in all sauropods bore the greater part of the body weight (Alexander, 1985, 1989). The proximal limb elements (humerus and femur) were distinctly longer than the lower limb bones. The metacarpus was arranged in a vertical semicircle (Bonnan, 2003), while the pes was semi-digitigrade. The feet probably bore a soft heel pad, like in modern elephants, as indicated by the extensive sauropod footprint record. The toes are reduced, or at least short. The rough and pitted articular surfaces of the long bones indicate thick cartilage caps around the major joints, which was recently confirmed by the fossilized remains of such a cap (Schwarz, Wings & Meyer, 2007*d*). Since the exact thickness and shape of the articular cartilage is not known and the range of motion of most limb joints is less easily constrained than in mammals, biomechanical analyses of sauropod locomotion are less precise than in mammals.

The trunk was short and deep. Characteristic of sauropods are the elongated pedicels of the vertebral arches (Upchurch *et al.*, 2004). This is a biomechanical adaptation to the statics of a long body stem, which is supported by two pairs of limbs, which are placed close together. Thus the bending moments produced by the weight of the body are “positive” (dorsally convex) along the full length of the trunk, and dorsal tension-resisting structures like muscles and ligaments are permanently stressed. A long distance between muscles and centra (= lever arm), which is provided by the long pedicels, reduces the forces that act along the body axis. This results in energy savings for the dorsal musculature and less substance and thus less weight for the vertebral bodies (Preuschoft, 1976).

Whereas sauropod limbs contained massive bones, their presacral vertebrae were a marvel of lightly constructed lamina systems (Osborn 1899; Janensch, 1929*a*; Wilson, 1999). With most of the weight being carried by the hindlimbs, the number of sacral vertebrae and thus the bony connection between the limbs and the vertebral column increased during sauropod evolution, from three sacrals in basal sauropodomorphs, to four in basal sauropods, five in most sauropods, and finally six sacral vertebrae in some titanosaurs (Wilson & Sereno, 1998; Wilson, 2002; Upchurch *et al.*, 2004). In contrast to most older illustrations and skeletal mounts of sauropods, osteology indicates that tail was held clear off the ground (Figs 1, 5), consistent with the lack of tail drag marks in sauropod trackways (Lockley, 1991).

The elongation of the neck involves both the elongation of single vertebrae as well as an increase in the total number of cervical vertebrae (up to 19 in *Mamenchisaurus*), which happened independently in several lineages. With the exception of brachiosaurid and probably camarasaurid and titanosaur sauropods, the long neck appears to have been held horizontally or slightly curved up when inactive. The long tail was crucial as a counterbalance during neck movements.

Smaller sauropods such as the Dicraeosauridae appear to have relatively shorter necks than the larger forms, and strongly positive interspecific allometry of neck length was found by Parrish (2006) for sauropodomorphs in general. Senter (2007), on the other hand, found no correlation between limb length and neck lenght in a sample of eleven sauropod taxa, probably because of the smaller sample size than that of Parrish (2006). Where it is known from ontogenetic series, juveniles have relatively shorter necks than adults, e.g. in *Camarasaurus* and Diplodocidae (Britt & Naylor, 1994; Ikejiri, Tidwell & Trexler, 2005; Schwarz *et al.*, 2007*b*). Such a positive intraspecific allometry of neck length is also seen in the basal sauropodomorph *Massospondylus* (Reisz *et al.*, 2005), in which the embryos have a relatively much shorter neck than the adults.

The long necks of sauropods (Fig. 5) are either interpreted as a means for high browsing (e.g. Bakker, 1987; Paul, 1987, 1998) or for increasing the horizontal feeding range (e.g. Martin, 1987; Mateus, Maidment & Christiansen, 2009; Seymour, 2009*a*, Preuschoft *et al.*, in press). Possibly, different species employed different feeding strategies (e.g. Dodson, 1990; Dzemski & Christian, 2007). This assumption is supported by ecological considerations and by the diversity in jaw and tooth morphology (e.g. Upchurch & Barrett, 2000; Sereno & Wilson, 2005). The neck posture of some of the largest forms, especially *Brachiosaurus* and its close relatives, is the subject of much controversy (Martin, 1987; Paul, 1998; Stevens & Parrish, 1999, 2005*a*, *b*; Seymour & Lillywhite, 2000; Christian, 2002; Berman & Rothschild, 2005; Dzemski & Christian, 2007; Christian & Dzemski, 2007, in press; Sereno *et al.*, 2007; Sander, Christian & Gee, 2009; Seymour, 2009*a*, *b*; Taylor, Wedel & Naish, 2009; Christian & Dzemski, in press). For *Brachiosaurus*, the suggested neck posture differs between horizontal (Stevens & Parrish, 2005*a*, *b*) and nearly vertical (e.g. Janensch, 1950; Paul, 1988; Christian & Heinrich, 1998; Christian, 2002). Sauropods probably employed different neck postures during different activities, like feeding, locomotion and standing at rest, so that reconstructions of neck postures can differ due to different approaches used for the reconstruction (Christian & Dzemski, 2007, in press; Dzemski & Christian, 2007).

Recent work (Christian & Dzemski, 2007, in press; Dzemski & Christian, 2007; Taylor *et al.*, 2009) indicates that the mobility of sauropod necks was underestimated by earlier studies (Stevens & Parrish, 1999, 2005*a*, *b*). Feeding over a large volume (the ‘feeding envelope’) was possible even if browsing was restricted by neck position to medium heights. In any case, the long necks of sauropods allowed them to feed not only at heights out of reached of other herbivores, but also over a large volume without moving the massive body.

The extensive sauropod trackway record potentially will inform us on the issue of the habitual neck position. Sauropod trackways always show much larger pes prints than manus prints, and the pes prints are more deeply impressed (Thulborn, 1990; Lockley & Meyer, 2000; Wright, 2005), indicating that most of the body weight was carried on the hindlimbs. This appears inconsistent with a horizontally held neck which would exert considerable leverage on the front limbs, necessitating larger feet and resulting in deeper imprints than observed, while this leverage would be much reduced if the neck were held more erect, consistent with the small and shallow manus prints. An in-depth review of the controversial neck position of sauropods is beyond the scope of this paper.

### (2) Musculature reconstruction and locomotor evolution

Osteological correlates of muscles and tendons, combined with comparative work in birds and crocodiles using the extant phylogenetic bracket approach, allow reasonably reliable reconstructions of musculature and its evolution. Applied to the limb musculature, such work (Remes, 2006, 2008; Fechner, 2009; Rauhut *et al.*, in press) leads to a deeper understanding of the musculoskeletal (and therefore biomechanical) design of the sauropod locomotory apparatus and its evolution. In the forelimb, a change from an adductor-driven to an abductor-driven locomotory system took place at the base of the Sauropoda (Remes, 2008*a*; Rauhut *et al.*, in press), while the same evolutionary change had already occurred at the base of the Dinosauria in the hindlimb (Fechner, 2009).

In contrast to mammals, sauropods retained the primitive condition of the caudofemoralis musculature as the main propulsive muscle in locomotion (Gatesy, 1990). In comparison to other archosaurs, the attachment area of this muscle is more distally placed, thus trading torque for increased lever arm length, clearly an adaptation to giant size. While the reconstruction of other parts of the sauropod musculature may bear on the issue of their unique gigantism, such work is still in its early stages.

### (3) Locomotion: gait and speed

Biomechanical calculations indicate that the large size of sauropods limited them to certain gaits, excluding the possibility of running, i.e. a gait with a suspended phase. Also, the extremely posterior position of the centre of mass in some groups (e.g. diplodocids) induces strong lateral moments during leg retraction that must be countered in the forelimbs. This must have made pacing and other gaits impossible in which the contralateral forelimb to the currently propelling hindlimb is protracted. In a walk, travelling speeds of 5.4–8.6 km h^−1^ were calculated on the basis of strictly pendulous, non-muscle-powered movements of the limbs (Preuschoft *et al.*, in press). Top speeds of nearly 20 km h^−1^ appear possible based on preliminary computer-aided engineering (CAE) modeling (Mallison, in press *b*). Sauropods are similar in their limb design to elephants, with sturdier sauropods having similar, or even slightly greater, strength indicators to extant proboscideans (Alexander, 1985). This indicates that they were comparably athletic. Since elephants can move at speeds of up to 35 km h^−1^ (Hutchinson *et al.*, 2006), we must assume that similarly sized sauropods achieved similar speeds, while larger animals with equal strength indicators were even faster.

The track record indicates that sauropods usually progressed at slow speeds (Thulborn, 1990; Christiansen, 1997; Lockley & Meyer, 2000), with estimates from trackways ranging from about 2 to 7 km h^−1^ (Thulborn, 1990; Mazzetta & Blanco, 2001). The average speed seems to have been below 2 km h^−1^. Faster locomotion might rarely have been recorded because a soft, sometimes slippery surface that might preserve footprints is not the kind of substratum a graviportal animal would run on.

### (4) Integument

Limited evidence exists for the structure of the integument in sauropods. Carbonized skin remains of diplodocid sauropods indicates that their skin was covered by a mosaic pattern of non-imbricating scales (Czerkas, 1994; Ayer, 2000), and the same was obviously also true for other sauropods (Mantell, 1850; Upchurch, 1995; Rich *et al.*, 1999). Along the dorsal midline, at least some of these animals sported a row of triangular skin flaps, probably serving display purposes (Czerkas, 1994). In titanosaurs, the skin additionally contained osteoderms (Upchurch *et al.*, 2004; Le Loeuff, 2005). Such a skin structure with a mosaic of scales is also seen in embryonic titanosaurid sauropods from Argentina (Chiappe *et al.*, 1998; Coria & Chiappe, 2007), which lack any indication of insulating structures to cover the naked skin.

### (5) Respiratory system

Sauropods are characterized by a dorsally placed, paired or unpaired bony narial opening which traditionally has been equated with fleshy nostrils in the same dorsal position. However, Witmer (2001) argues convincingly for far rostrally placed fleshy nostrils and a complex narial aparatus that may have improved heat exchange between air and blood stream. Based on palaeoneurological studies (Knoll, Galton & López-Antoñanzas, 2006), there is no evidence for the proboscis-like structure hypothesized by Bakker (1986), a conclusion reached earlier by Barrett (1994), Upchurch (1994), and Upchurch & Barrett (2000) based on jaw mechanics and tooth wear.

Much of the axial skeleton, sometimes even including the ribs, was strongly pneumatized (Henderson, 2004; Wedel, 2005, 2007, 2009; Schwarz, Frey & Meyer, 2007*a*), with pneumatization moving gradually backwards along the skeleton during sauropod evolution. In the most derived sauropods, it even invaded the tail vertebrae and the ischia. A consensus has recently emerged that this pneumatization indicates the presence of an avian-style flow-through lung and large airsacs in the body cavity of sauropods (Perry & Reuter, 1999; Wedel, 2005, 2009; O’Connor, 2009; Perry, Breuer & Pajor, in press). The same appears to have been the situation in theropods (O’Connor & Claessens, 2005; O’Connor, 2009) and thus must have evolved in the most basal saurischians, although the evidence for an avian-style respiratory system in basal sauropodomorphs is inconclusive (Wedel, 2007, 2009). Although a secondary hard palate is lacking in sauropods, some sort of fleshy folds must have been present that prevented food from entering the nostrils (Leahy, 2000), as in birds.

The bird-type lung would also have been advantageous in overcoming the problem of tracheal dead space caused by the very long trachea of sauropods (Perry, 1983, 1989; Daniels & Pratt, 1992; Calder, 1996; Hengst *et al.*, 1996; Paladino, Spotila & Dodson, 1997; Paul, 1998; Wedel, 2003*b*; Perry *et al.*, in press). In fact, in some birds such as swans—a group already with a long neck—the trachea makes an extra loop against the breast bone before it enters the body cavity (McLelland, 1989), indicating that dead space does not limiting tracheal length in the bird respiratory system.

### (6) Dentition and digestive system

All sauropods appear to have been exclusively herbivorous (Weishampel & Jianu, 2000; Upchurch & Barrett, 2000; Barrett & Upchurch, 2005; Stevens & Parrish, 2005*b*). However, recent finds (*Bonitasaura salgadoi*, Apesteguía, 2004; *Nigersaurus taqueti*, Sereno & Wilson, 2005; Sereno *et al.*, 2007) reveal an unexpected diversity of dentitions (Barrett & Upchurch, 2005), beyond the long-known distinction of pencil-shaped teeth restricted to the front of the snout in diplodocoids and titanosaurs *versus* the more massive dentitions of spoon-shaped teeth with wear facets in basal sauropods and basal macronarians (Sander, 1997; Upchurch & Barrett, 2005). This variety of dental designs can safely be assumed to reflect some degree of ecological niche diversification (Bakker, 1986; Calvo, 1994; Stevens & Parrish, 1999; Christiansen, 2000; Upchurch & Barrett, 2000, 2005). Furthermore, slight carbon isotope differences found in different sauropod taxa support a certain degree of niche partitioning or at least differences in dietary breadth and/or habitat (Tütken, in press). As large herbivores, sauropods must have relied on symbiotic gut microbes (contra Ghosh *et al.*, 2003), and their digestive tract must have contained capacious fermentation chambers, probably in the hindgut as in birds and herbivorous squamate reptiles (Farlow, 1987; Hummel *et al.*, 2008; Hummel & Clauss, in press). The evidence for fermentative digestion in sauropods consists of (*a*) phylogenetic bracketing that indicates that symbiotic fermentation bacteria were the same as in modern herbivorous birds and mammals, (*b*) all large recent herbivores employ fermentative digestion, and (*c*) the fact that sauropods would have needed to consume impossibly large amounts of plant matter without it (Hummel & Clauss, in press).

The food was gathered by shearing bites, nipping, or branch-stripping (Fiorillo, 1998; Barrett & Upchurch, 2005; Chatterjee & Zheng, 2005; Stevens & Parrish, 2005*b*). Because fermentation rate depends on particle size and the mastication capability of sauropods must have been rather limited, a gastric mill has long been hypothesized to serve in reducing plant particle size before fermentation. Occasional finds of polished pebbles with sauropod skeletons (e.g. Janensch, 1929*b*; Bird, 1985; Christiansen, 1996) were taken as evidence for such a gastric mill, but comparative and experimental work (Wings & Sander, 2007; Wings, 2007, 2009) on ostriches and other herbivorous birds indicates that these pebbles are not the remains of an avian-style gastric mill, leaving it uncertain whether and how sauropods reduced particle size.

Coprolites could potentially provide information on particle size, and data from faeces are extensively used in animal nutrition studies (Udén & Van Soest, 1982; Fritz *et al.*, 2009). Although putative sauropod coprolites containing grass phytoliths and many other plant remains have been described from the latest Cretaceous of India (Ghosh *et al.*, 2003; Prasad *et al.*, 2005; Mohabey, 2005), their sauropod affinity is difficult to establish (Mohabey, 2005; Sander *et al.*, in press *a*). As putative sauropod gastric contents (Brown, 1935; Stokes, 1964; Bird, 1985) are no longer tenable (Ash, 1993; Sander *et al.*, in press *a*), there is currently no direct evidence on sauropod food and food processing.

Sauropods as herbivores were thus most similar to extant herbivorous reptiles, but differ from herbivorous birds in the apparent lack of a gastric mill, and from ornithischian dinosaurs (which were exclusively herbivorous) and herbivorous mammals in their lack of extensive mastication. However, the data of Clauss *et al.* (2009) on the relationship between particle size and retention time in extant animals and those of Franz *et al.* (2009) for scaling of gut contents show that sauropods could have compensated for the lack of particle reduction by an increased retention time (as already suggested by Farlow, 1987). Franz *et al.* (2009) also concluded that the digestive system did not place constraints on sauropod body size.

### (7) Circulatory system

The circulatory system has few osteological correlates. However, aspects of the circulatory system of sauropod dinosaurs have received considerable attention, especially in conjunction with the position of the neck and blood pressure problems associated with it (Kermack, 1951; Badeer & Hicks, 1996; Seymour, 1976, 2009*b*; Seymour & Lillywhite, 2000; Gunga *et al.*, 2008; Ganse *et al.*, in press; review in Alexander, 2006). Phylogenetic bracketing (Witmer, 1995) and physiological arguments suggest that all dinosaurs had a four-chambered heart with a complete separation of pulmonary and body blood (Seymour, 1976; Paladino *et al.*, 1997; Gunga *et al.*, 1999; Seymour & Lillywhite, 2000) and, thus, were able to generate the blood pressure necessary to supply the brain in a raised head with blood. However, some researchers have argued that no sauropod would have been able to hold the neck upright habitually because of the very high blood pressure required which would damage the arterial tissue and also the brain if the sauropod ever were to lower its head (Choy & Altmann, 1992). These arguments do not take into account hypothetical soft tissue structures (Badeer & Hicks, 1996; Ganse *et al.*, in press) such as a rete mirabile (Colbert, 1993) or hypertrophied cardiac and arterial structures, which could have served to ensure an adequate supply of blood to the brain at a minimum energetic cost, as is seen in giraffes (Mitchell & Skinner, 2009). Some suggestions of hypothetical soft tissue structures appear exaggerated and are untestable, however, such as the presence of seven additional hearts along the neck (Choy & Altmann, 1992)—a structure unknown from all extant vertebrates. In sauropods that held the neck high, the heart must have been extraordinarily large to supply the head with blood (Seymour, 1976, 2009*b*).

### (8) Nervous system and sense organs

Some aspects of the central nervous system are accessible to palaeontological investigation because it has distinct osteological correlates, such as an ossified brain case and the neural canal of the vertebrae. Most recently, growth marks in dentine have also been used to infer characteristics of the nervous system (Appenzeller *et al.*, 2005).

Endocasts indicate that the brain of sauropods was small (Janensch, 1935–36; Jerison, 1969, 1973; Hopson, 1977, 1979; Knoll *et al.*, 2006) and not very highly developed (e.g. Chatterjee & Zheng, 2005). However, although the brain of sauropods was often said to be extraordinarily small, it actually falls within the allometric regression for a reptile of this size (Hopson, 1979). Boundaries between individual parts of the brain are often only poorly defined in available endocasts (e.g. Osborn, 1912; Osborn & Mook, 1921; Janensch, 1935–36; Hopson, 1979), indicating that the braincase was only partially filled by the brain, which was cushioned by connective and fat tissues, as in modern reptiles (Hopson, 1979). Most sauropods have a pronounced, tapering dorsal process over the cerebral hemispheres, which was interpreted as a parietal organ by some authors (Janensch, 1935–36), but an interpretation as an unossified zone or enlarged cerebral blood vessel seems to be more likely (Hopson, 1979). The pituitary gland seems to have a positive allometric relationship with body size and is thus very large in sauropods (Edinger, 1942).

Recently, Clarke (2005) studied in detail the vestibular labyrinth of *Brachiosaurus brancai* from Tendaguru, already described by Janensch (1935–36). The dimensional analysis of the labyrinth showed that body mass and the average semicircular dimensions of *Brachiosaurus brancai* generally fit with the allometric relationship found in previous studies of extant species. Most remarkable was that the anterior semicircular canals were found to be significantly larger than the allometric relationship would predict. Therefore Clarke (2005) hypothesized a greater sensitivity of the organ, which can be interpreted in a further step as slower pitch movements of the head in this direction, and most likely a flexion of the neck, rather than a head pitching about the atlas joint. These suggestions are supported by the most recent studies on the neck and head posture of *Brachiosaurus brancai* by Christian & Dzemski (2007; see also Dzemski & Christian, 2007). Semicircular canal arrangement also indicates the habitual pose of the head, from sligthly tilted upwards in prosauropods to horizontal in *Camarasaurus*, and increasingly downturned in diplodocoids, as recently described in the extreme form *Nigersaurus* (Sereno *et al.*, 2007).

Sauropods seem to have had large eyes (for their skull size) since sclerotic rings indicate that almost the entire orbit was filled by the eyeball, in contrast to larger theropods with similar skull sizes, in which the eyeball only occupied the dorsal part of the orbit. Sauropod nares are large, and the fleshy nose was obvioulsy a highly sophisticated structure (Witmer, 2001). Furthermore, the olfactory bulbs were well developed in sauropods (Janensch, 1935–36), indicating that olfaction was important to these animals.

An enlargement of the neural canal in the sacral region of sauropods has popularly been interpreted as a ‘second brain’. However, this enlargement, which can be considerably larger than the braincase of sauropods (Janensch, 1939), was probably filled with other tissues, such as a glycogene body as in modern birds (Giffin, 1991), and by nerves extending from the spinal cord to the legs.

Appenzeller *et al.* (2005) studied frequency domains and power spectra in growth marks in dentine of *Brachiosaurus brancai* teeth to assess the influence of the sympathetic (low frequency) and parasympathetic (high frequency) autonomic nervous system drive on the formation of this biological structure. The growth marks can be regarded as expressions of rhythmic falls and rises in blood supply to developing enamel and dentine. Blood supply, in turn, is controlled by the autonomous nervous system. In *Brachiosaurus brancai* low frequency bands indicate an active sympathetic nervous system which is consistent with the high hydrostatic pressures which the cardiovascular system would have had to overcome to ensure an adequate blood supply, especially to the brain.

### (9) Organ size and its scaling

Based on body mass estimates, allometric scaling equations (Calder, 1996; Schmidt-Nielsen, 1984) allow estimates of the size of various anatomical (skeletal mass, organ size, blood volume *etc*.) and physiological features (Schmidt-Nielsen, 1984). Applied to dinosaurs (Gunga *et al.*, 1995, 1999, 2002, 2007, 2008; Franz *et al.*, 2009; Ganse *et al.*, in press), these estimates are important in the modeling of many life functions of sauropods, such as growth, metabolism, respiration, locomotion, and reproduction. Not surprisingly, staggering values for body mass also result in staggering size estimates for organs, e.g. a 200 kg heart for a 38 t *Brachiosaurus* (Gunga *et al.*, 2008; Ganse *et al.*, in press). These estimates might also help to test other hypotheses as well, such as questions about tissue density and the size of organs. The latter can be derived from the body mass and calculated using scaling equations. Examples are the integument, respiratory system, heart, and gastrointestinal tract. It can then be tested whether these organs are actually anatomically able to fit into the thoracic and abdominal cavity of a sauropod. This has been attempted recently for *Brachiosaurus brancai* and especially *Plateosaurus engelhardti* (Gunga *et al.*, 2007, 2008; Franz *et al.*, 2009, Ganse *et al.*, in press).

### (10) Physiology and thermoregulation

Among the most debated aspects of sauropod biology (and of dinosaurs in general) is their metabolic rate (e.g. Seymour, 1976; Spotila *et al.*, 1991; Sander & Clauss, 2008), and this topic requires a somewhat more extensive treatment, beginning with the clarification of terminology. *Ectothermic* refers to an animal acquiring the heat necessary for the organism to function from the environment, while an *endothermic* animal generates this heat metabolically. *Poikilothermic* refers to an animal in which body temperature tracks ambient temperature, while *homoiothermic* refers to a constant body temperature that is elevated above ambient temperature. *Bradymetabolic* indicates the low basal metabolic rate (BMR) of most extant reptiles (∼30 kJ/kg body mass^0.75)^, while *tachymetabolic* refers to the elevated BMR seen in modern placental mammals (289 kJ/kg body mass^0.75^). As a general rule, a tachymetabolic animal has a BMR an order of magnitude greater than a bradymetabolic animal of the same body mass (Case, 1978; Schmidt-Nielsen, 1984, 1997; Walter & Seebacher, 2009).

#### (a) Lines of evidence

Evidence from bone histology, posture, ecology, oxygen isotope composition of skeletal apatite, and modeling has been used in elucidating dinosaur, including sauropod, thermometabolism, with contrary views having been advanced (for reviews, see Padian & Horner, 2004; Chinsamy & Hillenius, 2004). Arguments proposed for tachymetabolic endothermy include: the mammal-like posture of sauropods with their fully erect stance and gait (Ostrom, 1970) and the cardiac requirements resulting from this bauplan (Seymour, 1976), predator-prey ratios of dinosaur faunas (Bakker, 1975), fibrolamellar and Haversian bone tissue which is only seen in large mammals and birds today (de Ricqlès, 1980), much higher growth rates than in ectotherms as indicated by fibrolamellar bone and growth mark counts (Case, 1978; see below), low intra-bone oxygen isotope variability similar to endothermic mammals (Barrick & Showers, 1994; Barrick, Stoskopf & Showers, 1997), and latitude-dependent differences in enamel oxygen isotope compositions between sympatric ectotherms (crocodiles and turtles) and saurischian dinosaurs (Fricke & Rogers, 2000; Amiot *et al.*, 2006).

Bradymetabolic ectothermy appeared perhaps most strongly supported by modeling of heat exchange with the environment, indicating that a tachymetabolic sauropod would overheat (Dunham *et al.*, 1989; Spotila *et al.*, 1991; Alexander, 1989, 1998: O’Connor & Dodson, 1999). In fact, African elephants are said to be at the body size limit for tachymetabolic endotherms because of heat loss problems, being prone to heat stroke. Their large ears are major heat dissipation devices, raising the question of how an endothermic sauropod would have circumvented this problem. As already noted by Colbert (1993), because of their long necks and tails, sauropods had a much more favorable (i.e. higher) surface to volume ratio than a sauropod-sized elephant.

Overheating problems again are cited by a new study (Gillooly, Allen & Charnov, 2006) that combines a recent gigantothermy model with an avian-like gas exchange model that takes bradymetabolism into account. According to this model, a sauropod heavier than 10 tonnes would encounter body temperatures that are incompatible with life unless some cooling mechanism existed. However, the modeling results of Gillooly *et al.* (2006) may be compromised by their use of unrealistically high growth rates of >5000 kg year^−1^ for sauropods (Sander *et al.*, in press *b*). In addition, the tracheal surface and air sac system probably present in sauropods could have served as an efficient internal cooling system (Wedel, 2003*b*, Sander & Clauss, 2008; Perry *et al.*, 2009, in press) to prevent overheating during exercise and in high ambient temperatures.

Another argument for ectothermy was the scaling of foraging time (Midgley, Midgley & Bond, 2002), based on the observation that elephants feed 80% of their time. This comparison would suggest that an endothermic sauropod would not have been able to gather enough food due to time constraints. However, this argument is not valid (Sander & Clauss, 2008) because the time constraints encountered by elephants are due to their need to chew their food—which sauropods did not do—and their poor digestive efficiency (Clauss *et al.*, 2003*b*).

Weaver (1983) argued that head size in sauropods was too small to take in enough food for an endothermic metabolism. This hypothesis was rejected by Paul (1998) and Christiansen (1999) based on a comparative analysis of muzzle width in sauropods and mammals. Farlow (1987) added another twist to the debate by suggesting that the heat generated by fermentation of food in the sauropod gut “may have been a significant source of thermoregulatory heat”. However, Clarke & Rothery (2008) analysed body temperature across a large variety of mammalian species and concluded that no general pattern of either increasing or decreasing body temperature with body mass among herbivores was evident. In addition, Clauss *et al.* (2008*a*) reviewed evidence from measurements of BMR of animals of different digestive types, finding that BMR is not reduced in herbivores to compensate for fermentation heat, and suggesting that fermentative heat was not important in sauropod thermoregulation.

#### (b) Bone histologic evidence

Bone histologic evidence for ectothermy in sauropods was seen in lamellar-zonal bone with lines of arrested growth (LAGs) in sauropod bone tissue (Reid, 1981). However, this work has been superseded by in-depth studies of sauropod long bone histology. These document the overwhelming abundance of fibrolamellar bone indicative of very high growth rates. The argument by Reid (1981) and others (Chinsamy & Hillenius, 2004; Chinsamy-Turan, 2005) has also been weakened by the recognition that LAGs are common in bones of mammals (Klevezal, 1996; Horner, de Ricqlès & Padian., 2000; Sander & Andrássy, 2006). Although fibrolamellar bone has repeatedly been described in recent wild alligators with moderate growth rates (Chinsamy-Turan, 2005; Tumarkin-Deratzian, 2007), this has not been documented in sufficient detail, such as high-magnification photomicrographs and polarized light images, to substantiate these claims.

Our current understanding is that perhaps the strongest evidence for metabolic rate in sauropods comes from the numerous and detailed bone histologic studies conducted by different groups (Rimblot-Baly, de Ricqlès & Zylbergberg, 1995; Curry, 1999; Sander, 1999, 2000; Erickson, Rogers & Yerby, 2001; Sander & Tückmantel, 2003; Sander *et al.*, 2004, 2006, in press *b*Erickson, 2005; Klein & Sander, 2008; Lehman & Woodward, 2008; Woodward & Lehman, 2009). These all report fibrolamellar bone tissue in the long bones of virtually all sauropods (Klein & Sander, 2008; Sander *et al.*, in press *b*). Laminar fibrolamellar bone unequivocally indicates bone apposition rates only seen in endothermic vertebrates today. This is in agreement with data from growth mark records that indicate body mass gains of a few tons per year. Such growth rates are not seen in any living ectotherm (Case, 1978) and cannot be reconciled with the BMR of modern bradymetabolic terrestrial vertebrates but point to tachymetabolic endothermy in sauropods, at least during the phase of active growth (Sander *et al.*, in press *b*).

Bone histologic evidence for endothermy also consists of the loss of developmental plasticity in the sauropodomorph lineage, i.e. there is a tight correlation between body size and ontogenetic age in sauropods and terminal body size is not variable within species (Sander & Klein, 2005; Klein & Sander, 2008; Sander *et al.*, in press *b*). In the basal sauropodomorph *Plateosaurus*, on the other hand, developmental plasticity was still present, but in combination with fibrolamellar bone. This may represent an early stage in the evolution of endothermy in sauropodomorphs (Sander & Klein, 2005). Bone histology also shows that evolutionary body size increase in sauropodomorphs from basal sauropodomorphs to large sauropods was brought about by a strong increase in growth rate for which the evolution of tachymetabolic endothermy may have been a prerequisite (Sander *et al.*, 2004; Sander & Klein, 2005).

#### (c) Scaling effects: gigantothermy and ontogenetic change

Scaling effects are of primary importance in the discussion of sauropod BMR because surface area increases with the second power but volume increases with the third power. While in modern small to medium-sized species, the two strategies of endothermy and ectothermy are very distinctive, they may converge at very large body size due to scaling effects (Paladino , O’Connor & Spotila, 1990; Paladino *et al.*, 1997). This strategy, observed in the extant leatherback turtle, was termed gigantothermy by Paladino *et al.* (1990; see also Spotila *et al.*, 1991). As body size increases, BMR in reptiles, birds, and mammals increases with a slope of less than unity, with an exponent of either 0.67 (White & Seymour, 2003, 2005) or of 0.75 (Brody, 1945; Savage *et al.*, 2004) being found in the literature. The higher exponent, however, may not be real but may result from the increasing importance of heat production from fermenting gut contents in large herbivores (White & Seymour, 2005; Clauss *et al.*, 2008*a*).

Thus it has been recognized for some time that a fully grown sauropod dinosaur would not have been affected by the daily temperature cycle even if it was bradymetabolic (e.g Colbert, Cowles & Bogert, 1946; Alexander, 1989, 1998). Independently of BMR, adult sauropods must have been homoiotherms because of their very low surface to volume ratio, which meant that their body temperature would at best have fluctuated with the seasons, but not on a daily basis as in modern poikilotherms (Colbert, 1993; Seebacher, 2003).

The scaling effects discussed above apply to the changes in body size experienced by the individual during its ontogeny as well. However, these changes have received little attention so far. This is surprising because no other terrestrial vertebrate passes through five orders of magnitude during its ontogeny, from a juvenile of a BM of a few kg to a fully grown adult of >10 000 kg. The histologic growth record suggests that, at least from about 20% maximum linear size (Sander, 2000; Klein & Sander, 2008), juvenile sauropods grew at rates comparable to those of large mammals because they laid down the same type of laminar fibrolamellar bone. However, at a body size of 10^2^ kg, juvenile sauropods would not have enjoyed the benefits of gigantothermy and must have had the BMR of modern mammals. On the contrary, the heat flow models (e.g. Dunham *et al.*, 1989) indicate that up to a body mass of 10^3^ kg, juvenile sauropods faced the problem of excessive loss of metabolic heat if they did not possess some type of integumentary insulation such as feathers or hair. There is only one record of embryonic skin (Chiappe *et al.*, 1998) and none for juveniles. This embryonic skin appears naked, presenting a paradox.

#### (d) Synthesis

How can the evidence for tachymetabolism provided by bone histology be reconciled with the overheating problem indicated by heat exchange modeling of adult sauropods? As reviewed above, internal cooling surfaces must have existed that allowed sauropod dinosaurs to shed their excess body heat, and these presumably were located in the extensive air sac system and trachea of sauropods. The unique ontogenetic body size range of sauropods presumably was accompanied by an equally unique ontogenetic variation in BMR (Farlow, 1990; Sander & Clauss, 2008). Growing sauropod dinosaurs must have been tachymetabolic endotherms, but BMR may have decreased rapidly as maximum size was approached, when the heat loss problem became most severe, and a high BMR was no longer needed to sustain growth.

### (11) Life history, growth, and reproduction

The life of a sauropod began in an egg with a hard, calcareous shell. This is indicated by eggs with embryos of an indeterminate titanosaur from the Late Cretaceous locality of Auca Mahuevo, Argentina (Chiappe *et al.*, 1998, 2005; Salgado, Coria & Chiappe, 2005). Other Late Cretaceous localities around the world have yielded eggs and clutches of the same oogenus as the finds from Argentina (*Megaloolithus*) and presumably were laid by titanosaurian sauropod dinosaurs as well (Sander *et al.*, 2008; Griebeler & Werner, in press; Wilson *et al.*, 2010). Pre-Late Cretaceous sauropod eggs are unknown. Hard-shelled eggs of the basal sauropodomorph *Massospondylus* from the Early Jurassic of South Africa (Reisz *et al.*, 2005) suggest that all sauropodomorphs laid hard-shelled eggs. High shell porosity and field data from southern Europe and India indicate that most *Megaloolithus* clutches were buried in the substratum or under plant matter (Sander *et al.*, 2008). The exception is the eggs from Auca Mahuevo, Argentina, which show low porosity (Sander *et al.*, 2008; Jackson *et al.*, 2008) and probably were not buried.

Clutch size in the buried eggs was small (<10 eggs). None of the Late Cretaceous *Megalooltihus* eggs exceed 25 cm in diameter and 5 l in volume, which is extremely small compared to an adult sauropod. Small clutch size and size of the eggs suggests that several clutches were produced by the titanosaurid female per season, because otherwise parental investment would have been unrealistically small (Sander *et al.*, 2008; Griebeler & Werner, in press). Because of small egg size, sauropod hatchlings were also extremely small compared to the parent animals. This alone suggests that there was little parental care, and there is ample other evidence against parental care (Sander *et al.*, 2008; Myers & Fiorillo, 2009), with the possible exception of the Auca Mahuevo titanosaurs (Sander *et al.*, 2008). Thus, titanosaurid (and by extension, all other) sauropods produced numerous small eggs with very precocial young that were left to fend for themselves (Myers & Fiorillo, 2009) and suffered high mortality before reaching sexual maturity in the second or third decade of their life (Sander *et al.*, 2008). Sauropods differ fundamentally in this respect from terrestrial mammals which do not combine large body size with numerous small offspring, but show a negative correlation between the number of offspring and body size (Janis & Carrano, 1992).

Not only were hatchling sauropods very small compared to the adults, but they must have been very abundant in sauropod populations (Sander *et al.*, 2008; Griebeler & Werner, in press). Juvenile sauropods are rare finds and thus appear underrepresented in the fossil record (Carpenter & McIntosh, 1994; Foster, 2005), and only very few skeletons of small juveniles (less than 2 m in total length) are known (Schwarz *et al.*, 2007*b*). However, for most sauropod species known from several individuals, the material also represents growth series beginning at individuals less than half maximum size (see data in e.g. Sander, 2000; Klein & Sander, 2008; Sander *et al.*, in press *b*). Limited data from trackways and bonebeds suggest that sauropod herds were composed of a much higher proportion of juvenile animals than is observed in aggregations of mammalian herbivores (Paul, 1998; Myers & Fiorillo, 2009). Correspondingly, trophic energy represented by large herbivore species should have been available to a predator guild to a much higher degree in the sauropod ecosystem as compared to large mammal-dominated ecosystems with reproductive output of large herbivores confined to a few well-protected young (Hummel & Clauss, 2008).

Bone histology indicates that juvenile growth was very rapid because long bones of juveniles consist of highly vascularized fibrolamellar bone (Sander, 2000; Klein & Sander, 2008; Sander *et al.*, in press *b*) of the type seen in juvenile large mammals. The qualitative growth record also suggests that sexual maturity was reached well before maximum size (Sander, 2000; Klein & Sander, 2008; Sander *et al.*, in press *b*), a pattern that is consistent with other dinosaurs (Erickson *et al.*, 2007; Lee & Werning, 2008). Growth was determinate, as indicated by avascular bone with closely spaced growth marks in the outermost cortex (external fundamental system; Sander, 2000; Klein & Sander, 2008; Sander *et al.*, in press *b*).

Unlike in other dinosaurs (Erickson, 2005), growth rates have been difficult to quantify in sauropods because histologic growth marks are rare and appear late in ontogeny, if at all (Sander, 2000; Klein & Sander, 2008; Sander *et al.*, in press *b*). Compared to other dinosaurs, bone histology suggests that sauropods had the highest growth rates as evidenced by the lack of growth marks and the limited comparative data from growth curves (Erickson *et al.*, 2001). The few growth mark records that are available suggest that full size was reached in less than four decades (Curry, 1999; Sander, 1999, 2000; Sander & Tückmantel, 2003; Wings *et al.*, 2007; Lehman & Woodward, 2008; Sander *et al.*, in press *b*). Maximum growth rates in the exponential phase of growth may have ranged from 500 kg to 2000 kg per year (Wings *et al.*, 2007; Lehman & Woodward, 2008), and earlier estimates of over 5000 kg per year (Erickson *et al.*, 2001) are exaggerated (Lehman & Woodward, 2008; Sander *et al.*, in press *b*).

Similarly, age at sexual maturity is difficult to ascertain, but it seems to have occurred in the second or third decade of life (Sander, 2000; Sander & Tückmantel, 2003). Both of these estimates are maximal ages because growth rate must have been slower in the individuals with growth marks than in the majority of sauropod samples, which lack growth marks. Based on survivorship curves for large extant herbivores, Dunham *et al.* (1989) also argue for an age at first reproduction of less than 20 years in sauropods.

Adult sauropods presumably were almost immune from predation because of their body mass being an order of magnitude greater than that of the largest predators. Their sheer volume made it difficult for an attacker to place an effective bite rather than scratch the skin (Preuschoft *et al.*, in press). With sauropod hatchlings being so small, there must have been strong selection pressure for high juvenile growth rates because they would have shortened the time during which the young sauropods were endangered by predators. Selection for high growth rates would have been particularly strong without parental care. In more general terms, a high growth rate fueled by a high BMR is a prerequisite for giant body size because tetrapods with a low BMR grow too slowly to benefit from the selective advantages of large body size. A high BMR thus emerges as a prerequisite for gigantism.

## III. BODY SIZE EVOLUTION IN SAUROPODOMORPHA

### (1) Body size in basal dinosauriforms and basal sauropodomorphs

In order to understand the evolution of gigantism in sauropods, it is necessary to consider the body sizes of both the immediate (basal sauropodomorphs) and more remote outgroups (basal saurischians, basal dinosauriforms) to Sauropoda.

The oldest and most basal dinosauriforms are found in the Middle Triassic of Argentina (Novas, 1996) and include animals such as *Marasuchus* (Sereno & Arcucci, 1994) and *Pseudolagosuchus* (Arcucci, 1987). These dinosaurian ancestors were surprisingly small animals probably weighing less than 1 kg. There seems to be a size increase at the base of Saurischia, although most basal saurischians (e.g. *Eoraptor*, Sereno *et al.*, 1993; *Guaibasaurus*, Bonaparte, Ferigolo & Riberio, 1999) are still of moderate size, with an estimated body mass well below 100 kg, and maybe even less than 10 kg (see Peczkis, 1994, for a body mass estimate for *Eoraptor*; *Guaibasaurus* was of similar size). The same is true for the basalmost sauropodomorphs known, *Saturnalia* (Langer *et al.*, 1999), *Panphagia* (Martinez & Alcober, 2009), and *Pantydraco* (Yates, 2003; Galton, Yates & Kermack, 2007), although the latter is only known from juvenile individuals.

A notable size increase is seen within basal sauropodomorphs, but the phylogenetic uncertainty in this part of the dinosaur tree makes an interpretation of the evolution of body size difficult. However, many typical ‘prosauropods’, such as *Plateosaurus* from the Late Triassic of Central Europe and *Riojasaurus* from contemporaneous rocks of Argentina, reached masses well over 2 t (e.g. Sander, 1992; Peczkis, 1994), and fragmentary remains from various Late Triassic and Early Jurassic formations indicate that some of these animals might well have exceeded 4 t (Rauhut personal observation). Evolutionary size increase in ‘prosauropods’ was obviously not linear: based on the phylogenetic hypotheses of Yates (2004, 2007), one of the smallest known ‘prosauropods’, *Anchisaurus*, with an estimated mass of less than 50 kg (Peczkis, 1994), is more closely related to sauropods than several taxa that exceeded 1 t.

Fechner (2009, see also Rauhut *et al.*, in press) points out that increasing size was also an important, if not the most important, determinant in the evolution of dinosauriforms and that many osteological, myological, and functional characteristics of sauropod dinosaurs can only be understood by taking the evolution of basal dinosauriforms into account.

### (2) Body size in early and basal sauropods

Very large sauropod humeri (Buffetaut *et al.*, 2002) from the Triassic of Thailand document the very rapid evolution (within a few million years after their origin) of very large body size in sauropods (Buffetaut *et al.*, 2002; Sander *et al.*, 2004). This rapid body size increase resulted from an evolutionary increase in growth rate compared to relatively small basal sauropodomorphs such as *Plateosaurus* (Sander *et al.*, 2004). This increase in growth rate appears to be linked to the evolution of tachymetabolic endothermy in the sauropdomorph lineage (Sander *et al.*, 2004; Sander & Klein, 2005; Sander *et al.*, in press *b*; see also section II.10*b*).

Sauropods are apparently unique among dinosaurs because the other major dinosaur lineages (with the possible exception of Theropoda) show a gradual body size increase over tens of millions of years (Sander *et al.*, 2004; see also Hone *et al.*, 2005; Carrano, 2006). The Early Jurassic sauropods from India (*Barapasaurus* and *Kotasaurus*) also represent large forms, as do the Middle and Late Jurassic sauropods from China, e.g. *Mamenchisaurus* (Wings *et al.*, 2007), and other areas, such as the Patagonian *Patagosaurus* (Bonaparte, 1986). That theropods may also have evolved rapidly to very large size, is suggested by footprints left by *Allosaurus*-sized theropods in the Late Triassic (Thulborn, 2003; Lucas *et al.*, 2006) and the remains of an *Allosaurus*-sized coelophysoid from the Late Triassic of Bavaria, Germany (Rauhut, personal observation).

### (3) Body size in Neosauropoda

The Neosauropoda, all taxa more derived than the sauropods discussed in the previous section, are characterized by large to giant body size with a few notable exceptions, i.e. the repeated occurrence of island dwarfing (Sander *et al.*, 2006; Stein *et al.* in press; Benton *et al.*, 2010) and the apparent trend in some titanosaurs towards evolutionary body size reduction with no apparent island effects (Hone *et al.*, 2005; Carrano, 2005, 2006). However, some of the largest sauropods also evolved among the Titanosauria (Bonaparte & Coria, 1993; Novas *et al.*, 2005; Calvo *et al.*, 2007). Within diplodocoids, the dicraeosaurids are also characterized by relatively small body sizes (Rauhut *et al.*, 2005). However, no truly small sauropods are known. Even the ‘dwarf’ sauropods were animals with an adult body mass well in excess of 500 kg (Peczkis, 1994; Sander *et al.*, 2006; Stein *et al.*, in press), a size which is reached by less than 10% of modern mammal species (Hotton, 1980).

### (4) Independent gigantism in several lineages

**Fig. 6:**
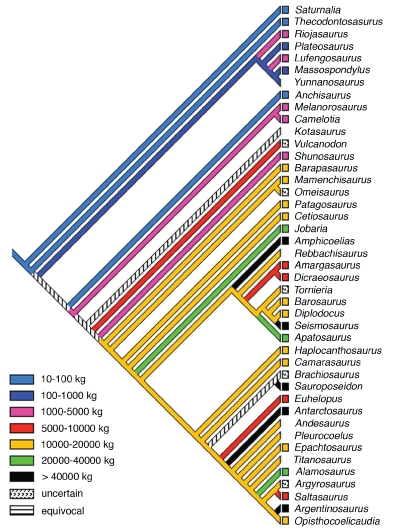
Independent evolution of gigantic species (>40 t body mass) in several lineages of Sauropoda as shown by optimization of body size on a sauropod phylogeny (part of the supertree of Dinosauria published by Lloyd *et al.*, 2008). Note that *Turiasaurus*, *Paralititan*, *Puertasaurus*, *Futalognkosaurus*, and *Huanghetitan* are not listed because they were not covered by this phylogeny. Body masses were taken from various sources (see Table 1). Lack of a colored box in front of the genus name indicates a lack of mass data.

Although sauropods were large animals in general, it is important to point out that extreme sizes (close to or in excess of 40 t) were reached independently by several different lineages of sauropods at different times throughout the later Mesozoic (Fig. 6). Specific cases are the Late Jurassic (Kimmeridgian) basal eusauropod *Turiasaurus* (Royo-Torres *et al.*, 2006), possibly the basal diplodocoid *Amphicoelias* (Carpenter, 2006), the Late Jurassic (Tithonian) *Diplodocus (Seismosaurus) hallorum* (Gillette, 1991, 1994; Herne & Lucas, 2006) and ‘*Supersaurus*’ (Upchurch *et al.*, 2004) among the Diplodocoidea, the Early Cretaceous (Aptian) brachiosaurid *Sauroposeidon* (Wedel *et al.*, 2000*a*, *b*), and several titanosaurs. The latter include *Paralititan* from the early Late Cretaceous (Cenomanian) of Egypt (Smith *et al.*, 2001), as well as *Argentinosaurus* (Bonaparte & Coria, 1993; Mazzetta *et al.*, 2004), *Puertasaurus* (Novas *et al.*, 2005), *Antarctosaurus giganteus* (Van Valen, 1969; Mazzetta *et al.*, 2004), and *Futalognkosaurus* (Calvo *et al.*, 2007) from the Late Cretaceous of Argentina. Extreme size among these very large titanosaurs probably evolved independently as well, but this is difficult to evaluate because of the uncertain relationships of these taxa within Titanosauria. Other examples of independent evolution of gigantism in sauropods may include the poorly known *Huanghetitan ruyangensis* from the middle Cretaceous of China, which has ribs over 3 m in length (Lü*et al.*, 2007).

Giant sauropods thus occurred from the Late Jurassic to the Late Cretaceous, over a time span of at least 85 million years, and this extreme gigantism developed independently in most major groups of neosauropods (Fig. 6). The large number of very recently described giant forms suggests that truly giant forms may have been even more common than suggested by the current fossil record. The stratigraphic range of these extreme giants and the fact that some of the largest sauropods are found in the latest Cretaceous (e.g. *Puertasaurus*) are especially noteworthy in light of the size-area relationship outlined by Burness *et al.* (2001), since their gigantism thus does not seem to be influenced by the progressice fragmentation of the supercontinent of Pangea during this time.

Differences in skull morphology, neck anatomy and reconstructed neck position in the different lineages that evolved giant sauropods indicate different feeding types (Upchurch & Barrett, 2000; Barrett & Upchurch, 2005). This suggests adaptations other than a specific feeding mode lead to very large body size.

### (5) Island dwarfing

As best exemplified by Quaternary proboscideans (Roth, 1990, 1992; Guthrie, 2004; Vos, Van den Hoek Ostende & Van den Bergh, 2007), dwarfed taxa of large to giant herbivores may evolve in island situations. The diminutive latest Cretaceous titanosaur *Magyarosaurus* from Romania has long been considered to have been an island dwarf (Nopcsa, 1914; Weishampel *et al.*, 1991; Jianu & Weishampel, 1999; Benton *et al.* 2010), but only the study of bone histology provides unequivocal evidence for island dwarfing. This was the case in the Late Jurassic basal macronarian *Europasaurus* (Sander *et al.*, 2006), but *Magyarosaurus* has now passed the test as well (Benton *et al.*, 2010; Stein *et al.*, in press). Other instances of putative island dwarfing are sauropods from the Albian-Cenomanian Adriatic-Dinaric carbonate platform (Dalla Vecchia, 2005). The latest Cretaceous titanosaurs *Rapetosaurus* from Madagascar and *Ampelosaurus* from southern France and northern Spain may also represent island forms (as already noted for *Ampelosaurus* by Jianu & Weishampel, 1999), because southern France together with the Iberian peninsula also formed a large island (Dercourt *et al.*, 2000) and Madagascar had already split from mainland Africa at this time (Smith, Smith & Funnell, 1994). Another example of a larger island dwarf may be represented by the Late Jurassic *Cetiosauriscus* from Switzerland (Schwarz, Meyer & Wings, 2007*c*).

The estimated body mass of *Europasaurus* was around 800 kg (Stein *et al.*, in press), and that of *Magyarosaurus* was in the same range (700–1000 kg, Peczkis, 1994). These island dwarfs are informative for the evolution of body size changes in sauropods because they show that the decrease in body size evolved through a decrease in growth rate (Sander *et al.*, 2006; Stein *et al.*, in press), the reverse of what is seen in the evolutionary increase in growth rate leading to the first large sauropods (Sander *et al.*, 2004).

### (6) Body size evolution and Cope's Rule

Two independent studies (Hone *et al.*, 2005; Carrano, 2006; see also Carrano, 2005) have recently attempted to quantify body size evolution in dinosaurs, and in sauropods in particular, to assess whether Cope's Rule was in operation in these groups. Using phylogenetically independent comparisons, Hone *et al.* (2005) showed that there was a strong but gradual body size increase in Dinosauria as a whole, while Sauropoda showed a rapid size increase in the Triassic but apparently decreased in size during the Cretaceous. Carrano (2006) evaluated body size change in all of Dinosauria and in major subclades such as Sauropodomorpha using squared change parsimony. He concluded that dinosaurs in general, as well as most subclades, showed a continuous increase in body size during their evolutionary history. Exceptions were the Sauropodomorpha, where in Macronaria there appeared to be a reduction in body size represented by several small-bodied titanosaurs, and the Theropoda (Carrano, 2006). Both these studies, however, are superseded by the new finds of giant titanosaurs and the island dwarfs reviewed above, and Carrano (2006) also did not include some large-bodied titanosaurs such as *Alamosaurus*.

Our current understanding would suggest that Macronaria in general and Titanosauria in particular extended the ancestral body size range to relatively very small and very large forms, and the giant South American Cretaceous sauropods (*Argentinosaurus*, *Antarctosaurus*, *Puertasaurus*, *Futalognkosaurus*) appear particularly ‘oversized’ for the landmasses they were inhabiting. While Carrano (2006) was not able to offer an explanation for the more numerous relatively small titanosaurs, a closer look at palaeogeographic change from the Middle Jurassic to the end of the Cretaceous combined with the area-body size relationship established by Burness *et al.* (2001) does: we observe that both the breakup of Pangea and the sea level rise since the Triassic resulted in a fragmentation of land masses and an increased number of islands. With sauropod body size, as the largest inhabitants of the land masses, being closely tied to land mass size (Burness *et al.*, 2001), the evolution of smaller forms was the result. The island dwarfs *Europasaurus* and *Magyarosaurus*, being the smallest macronarian sauropods, are only the most extreme known results of this process. Other dinosaur lineages continued to increase in average size until the end of the Cretaceous (Hone *et al.*, 2005; Carrano, 2006), despite the ever-increasing fragmentation of land masses, because they had not reached the upper limits of body size for the landmass they were inhabiting.

## IV. HYPOTHESES EXPLAINING GIANT BODY SIZE

### (1) Limits to body size

Given that Cope's Rule in its most general formulation is valid (Bonner, 2006), the question with regard to sauropod dinosaurs must be what limited their body size (Alexander, 1989), not what drove body size increase. The existence of limits to body size in the extant fauna is underscored by the body size-land area relationship of Burness *et al.* (2001). Sauropods (and theropods) somehow circumvented the constraints imposed on mammals and other dinosaurian groups (Carrano, 2006), raising the question of the nature of these constraints. For heuristic purposes, we will repeatedly ask how these constraints act on mammalian megaherbivores (defined as herbivores exceeding 1000 kg body mass; Owen-Smith, 1988) and on large ground birds and draw a comparison between mammalian megaherbivores and sauropod dinosaurs. The question of sauropod gigantism thus is linked throughout to the question of why other groups, most notably mammals, have not reached similar dimensions, even though they are well within the theoretical limits for terrestrial animals (Hokkanen, 1986). Although the subject will be touched upon repeatedly, exploring the theoretical limits of the tetrapod bauplan in the terrestrial realm is not the topic of our paper.

The constraints limiting body size fall into two broad categories: intrinsic constraints, founded in the animal's design and physiological makeup, and extrinsic constraints, founded in biotic and physical factors of the environment an animal inhabits, i.e. the boundary conditions of the system. As the example of gigantism in Carboniferous dragonflies illustrates (Beerling, 2007; Lighton, 2007), intrinsic and extrinsic constraints are mutually effective on an organism. Thus, because of design limitations of the tracheal respiratory system of dragonflies, in today's atmosphere of 21% O_2_ they are limited to a maximum body length of 12 cm and a wingspan of 16 cm. During the Carboniferous, the same biological design allowed wing spans of over 70 cm due to an oxygen level of 30% (Berner *et al.*, 2003; Berner, VanDenBrooks & Ward, 2007). When oxygen level fell in the Permian and Triassic (Berner, 2006; Berner *et al.*, 2007), dragonflies decreased in maximum body size (Beerling, 2007; Lighton, 2007).

Gravity also limits body size, and the current gravity constant of 0.981 ms^−2^ has been proposed to limit body size to 20 t (Economos, 1981) based on a mass estimate for the largest land mammal ever, *Paraceratherium* (also known as *Indricotherium*). However, sauropods were much heavier than the largest land mammals, and Günther *et al.* (2002) suggested the upper limit for terrestrial organisms due to gravitational forces to be at least 75 t. Similarly, Hokkanen (1986) calculated that bone strength and muscle forces only become limiting to terrestrial animal size at masses in excess of 100 t.

### (2) Resource availability

A different approach to understanding the limits of body size is resource availability. Resource availability has long been considered important in island habitats (Palkovacs, 2003) but, as suggested by the maximum body mass-land area relationship of Burness *et al.* (2001) (Fig. 3), it is of general importance for explaining the upper limits of body size. This constraint has its explanation in the relationship between resources available to the top species, its population density, and its risk of chance extinction (Janis & Carrano, 1992; Farlow, 1993; Paul, 1994, 1997*b*, 1998).

As each individual of the top species requires a certain amount of the available resources, expressed as its home range (Burness *et al.*, 2001), and resources are related to land area, the size of a landmass determines the number of home ranges and thus individuals of the top species that can inhabit it. The amount of resources required by an individual depends on its body size and its BMR. The larger and more metabolically active the individuals, the fewer are supported by the landmass. With increasing body size, the number of individuals with a given BMR will decrease, reaching a threshold below which chance extinction becomes increasingly likely. This is what, according to Burness *et al.* (2001), places an upper limit on body size, since all of the Earth's landmasses are of limited size and can be viewed as islands.

Land area is, of course, only a crude proxy for a population's resources which depends on the portion of the landmass that is actually inhabitable, on the productivity of an area, but also on intra- and interspecific competition for resources. According to the classical theory of island biogeography (MacArthur & Wilson, 1967), larger islands have more individuals per taxon (the authors assume a linear increase with increasing area size), which increases intraspecific competition for resources, and they also have more species, which increases interspecific competition.

However, in addition to land area (as a proxy for available resources) and BMR, there is a crucial third factor in the maximum body size-land area relationship which was not discussed by Burness *et al.* (2001). This factor is recovery rate after a severe population crash. It greatly influences the likelihood of chance extinction of the top species (Janis & Carrano, 1992; Farlow, 1993). Not surprisingly, high *per capita* resource availability and high population growth rates are factors known in conservation biology to increase the chance of population survival (Gilpin & Soulé, 1986; Primack, 1993). From an evolutionary perspective, high population recovery rates can also be viewed as an adaptation to overcome temporary resource limitations, because a species that has a high recovery rate can ensure its long-term survival, even under low population densities and thus on temporarily limited resources.

**Fig. 7:**
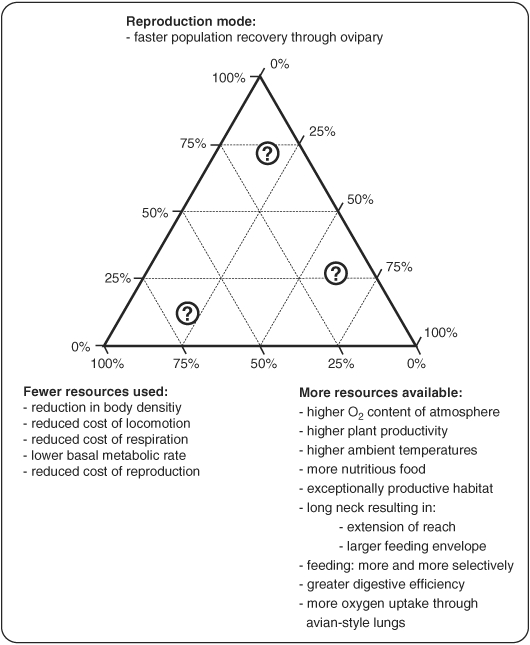
Three factors, i.e., more resources available, fewer resources used, and the reproduction mode, potentially resolved the land area *versus* body size enigma of Burness *et al.* (2001) and thus contributed to the gigantism of sauropods and theropods. Specific hypotheses (discussed in the text) underlying each contributing factor are listed below each factor. Because very likely more than one factor was important, the relative contribution of each is best visualized in a ternary diagram. The symbols with the question marks indicate potential solutions to the gigantism enigma, and the relative importance of each factor can be read off the percentage scale leading up to its respective corner. Note that we do not offer a final solution but that this graph is meant to visualize the possibilities of interplay between the three factors.

In the remainder of this paper, we will approach the gigantism issue from the resource perspective. This perspective takes all constraints into account and aids in formulating hypotheses about how sauropod dinosaurs overcame them (Fig. 7).

## V. MORE RESOURCES AVAILABLE THROUGH DIFFERENT BOUNDARY CONDITIONS

### (1) Physical boundary conditions

Although gravity is of overriding importance in determining the bauplan of an organism, we have to assume that there were no secular variations in Earth's gravity in the Phanerozoic geologic past (Economos, 1981). Among other possibly different boundary conditions, atmospheric oxygen levels (Hengst *et al.*, 1996; Berner *et al.*, 2007; Ward, 2006), levels of carbon dioxide (Maurer, 2002) as well as higher ambient temperatures have been implicated in sauropod dinosaur gigantism (Fig. 7).

#### (a) Increased oxygen content of atmosphere

**Fig. 8:**
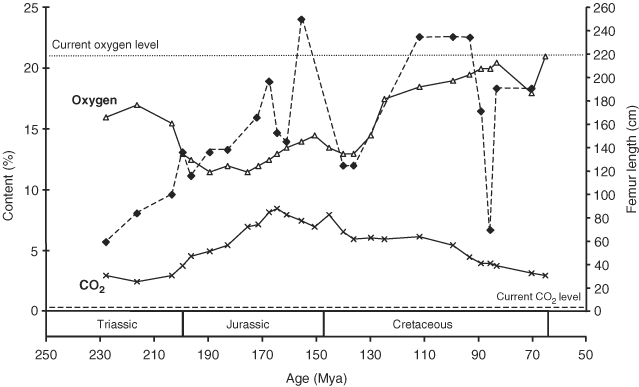
Variation of atmospheric composition(O_2_, CO_2_) and body size through time. Each data point is located at the beginning of a stage, starting with the Carnian and ending with the Cretaceous-Tertiary boundary. The variation of body size through time is an extension of the Carrano (2006) data set with femur length as a proxy for body size. Missing data points for body mass are either due to lumping of data from two stages (i.e. the Kimmeridgian and Tithonian) or missing data (i.e. for the Berriasian, Barremian, and Aptian). Body size increases gradually from the Late Triassic to the Late Jurassic, forming a plateau in the Cretaceous. The two sharp drops in body mass in the Early and Late Cretaceous are probably due to a poor terrestrial fossil record at these times. Note the lack of correlation between atmospheric composition and sauropod body mass. CO_2_content of the atmosphere also determines global temperature, and this graph thus suggests that sauropod body size is not correlated with global temperature variations through time, either. The data for O_2_ and CO_2_ levels are from Ward (2006).

All else being equal, would an increased level of atmospheric oxygen allow the evolution of gigantic terrestrial tetrapods? This possibility is suggested by the example discussed above of the uniquely gigantic dragonflies of the Carboniferous (Lighton, 2007). Hengst *et al.* (1996) explored this hypothesis for sauropod dinosaurs, based on the premise of an oxygen level of 30% or above in the Jurassic atmosphere (Landis *et al.*, 1996). Physically modelling respiration in the Late Jurassic sauropod *Apatosaurus*, they concluded that the respiratory system of this animal could not have delivered enough oxygen to the tissues at today's oxygen levels. This applied even under the assumption that *Apatosaurus* had the basal metabolic rate of a reptilian ectotherm. However, the hypothesis of Hengst *et al.* (1996) is superseded by the likely presence of a bird-like lung in sauropods and the current understanding that oxygen levels were significantly lower in the Jurassic and Cretaceous than today (Gans *et al.*, 1999; Dudley, 1998; Berner, 2006; Berner *et al.*, 2007; see also Fig. 8) or at about the same level (Bergman, Lenton & Watson, 2004; Belcher & McElwain, 2008).

#### (b) Increased plant productivity through increased CO_2_ content of the atmosphere

Another hypothesis that has been advanced is that an up to tenfold higher CO_2_ content of the Mesozoic atmosphere than today (e.g. Royer *et al.*, 2004; Berner, 2006; see also Fig. 8) increased plant productivity, thus allowing larger body size on the same plant resources (Paladino *et al.*, 1997; Burness *et al.*, 2001; Maurer, 2002). However, experiments have shown that while an increased CO_2_ level does increase plant productivity, the decrease in protein content and increase in levels of nonstructural carbohydrates and phenolics in sum result in a decreased food quality (at least for insects) (e.g. Roth & Lindroth, 1995; Ehleringer, Cerling & Dearing, 2002), thereby offsetting the hypothesized effect at least partially. Midgley *et al.* (2002) also question the increased plant productivity as a result of increased atmospheric CO_2_ because global primary productivity probably is saturated at much lower CO_2_ levels than those of the Mesozoic, arguing that water and nutrient availability were the limiting factors. In addition, while on average, CO_2_ level may have been much higher than today, it was by no means constant (Berner, 2004; Berner *et al.*, 2007). There is no obvious correlation of CO_2_ level with body size evolution in sauropods (Fig. 8), such as a sudden increase of CO_2_ concentration in the Late Triassic (to produce the first large sauropods), a high in the Kimmeridgian and Tithonian (to result in gigantism in several lineages of sauropods at this time), and in the Late Cretaceous (the age of the giant titanosaurs). Thus, we suggest that increased atmospheric CO_2_ levels were not a prerequiste to the evolution of gigantism in sauropods.

#### (c) Higher ambient temperatures

Higher average ambient temperatures are also worth considering because they would tend to blur the concepts of endo- *versus* ectothermy: with high ambient temperatures, homoiothermy can be achieved in large, compact animals with a low metabolic rate (Spotila *et al.*, 1991), whereas tachymetabolic species face overheating in the absence of effective physiological or behavioural cooling mechanisms. Thus, large size could provide the advantages of a high, constant body temperature at low cellular metabolic cost (gigantothermy: Dunham *et al.*, 1989; Spotila *et al.*, 1991; Paladino *et al.*, 1997).

A correlation between ambient temperature and maximum body size has been established for modern ectotherms (Makarieva *et al.*, 2005), and gigantism in snakes appears to be related to a global thermal maximum (Head *et al.*, 2009). Although not treated extensively in the recent scientific literature (but see Paladino *et al.*, 1997), the hypothesis that increased average ambient temperature during the Mesozoic greenhouse allowed the exceptional body size of sauropods needs to be considered from the point of view of the energy budget of a living sauropod. Higher ambient temperatures could have benefitted an endothermic animal in that less of the fodder taken in would need to be alotted to generating body heat (Seebacher, 2003). A poikilothermic dinosaur would have profited from increased ambient temperatures because of the ability to forage longer and more intensively, thus taking up more energy. However, the hypothesis of higher ambient temperatures permitting gigantism is not compatible with laboratory experiments on ectotherms (Atkinson & Sibly, 1997) and observations on endothermic mammals (Bergmann's Rule) and birds (reviewed by Ashton, 2001), because endothermic animals tend to be larger in colder environments and not *vice versa*.

As atmospheric temperature is generally believed to be determined by CO_2_ content (Wallmann, 2007), one again should look for a correlation between sauropod body size and atmospheric CO_2_ levels to test the hypothesis that extreme temperatures led to extreme body sizes of sauropods. However, this correlation is not apparent (Fig. 8).

### (2) Biological boundary conditions

#### (a) More nutritious food

Another extrinsic biotic hypothesis is that sauropod gigantism was made possible by some or all of the plant groups of the pre-angiosperm flora, such as cycads, ginkgoes, conifers, and ferns, being more nutritious than the plant groups preferentially ingested by modern herbivores, namely grasses and dicot leaf browse. Laboratory experiments designed to evaluate metabolizable energy content of the pre-angiosperm flora (Hummel *et al.*, 2008) show that several of these plant groups offer herbivores energy yields comparable to modern angiosperm browse (*contra*Weaver, 1983) while others had much lower yields. In particular, all three tested species of *Equisetum* offered high levels of energy, even reaching the level of grasses or herbs. *Araucaria* foliage also reached high levels, but only after prolonged fermentation (Hummel *et al.*, 2008). Non-podocarpaceous conifers, *Ginkgo*, and some ferns such as *Angiopteris* would also have yielded as much energy as the most nutritious food plants available to modern herbivores today (Hummel *et al.*, 2008). However, protein content of *Araucaria* was insufficient for this plant to have served as the sole food source of a growing sauropod (Hummel *et al.*, 2008). Thus, the sauropods' dietary choices, which were restricted to the pre-angiosperm flora before the mid-Cretaceous, apparently did not pose an obstacle in their evolution of gigantic body size nor did they foster it.

The two groups of food plants that offer the greatest amount of energy, *Equisetum* and *Araucaria*, most likely grew in large, monospecific stands, such as dense thickets around waterways (*Equisetum*) or in forests (*Araucaria*), much as they do today. They would have offered great amounts of biomass in a concentrated area to the continuously browsing sauropods. This also would have applied to other forest-forming plant groups such as the various families of conifers. Other plant taxa, for example, ferns, cycads, and bennettitites, were probably patchier or sparser in their distribution and thus less dependable as a food source (Gee, in press).

While some Mesozoic plants were both highly nutritious and abundant, there is no evidence from fermentation experiments to explain sauropod gigantism through more nutritious food. In addition, even if Mesozoic forage was more nutritious than modern forage and would have made gigantism possible, one would have to explain why this led to the unique gigantism of sauropods. In fact, herbivorous dinosaurs in general do not show any obvious evolutionary response to the rise of angiosperms (Coe *et al.*, 1987; Wing & Tiffney, 1987; Weishampel & Jianu, 2000; Barrett & Willis, 2001; Lloyd *et al.*, 2008; Butler *et al.*, 2009).

#### (b) Exceptionally productive habitats: mangroves and tidal flats

Other hypotheses based on increased resource availability from plants are those that involve exceptionally productive habitats. One such hypothesis is that of Smith *et al.* (in Nothdurft & Smith, 2001; see also Smith *et al.*, 2001), in which they recognize mangroves as allowing sauropod gigantism. This hypothesis was based on the discovery of the giant sauropod *Paralititan* from the Cenomanian of Egypt but was seen to be of general applicability. Based on associated plant remains (of the fern *Weichselia*), the authors concluded that *Paralititan* had preferentially inhabited mangrove environments. Mangroves, today being the second most productive environments on Earth after the tropical rainforests, would thus have provided the resource base for the evolution of exceptional body size. This hypothesis was based on the largely unsubstantiated premises that *Weichselia* is a mangrove plant, that such fern mangrove communities were very widespread and as productive in the past as modern angiosperm mangrove communities are today, and that *Paralititan* really inhabited these environments (Smith *et al.* in Nothdurft & Smith, 2001). While this hypothesis may be applicable to the case of *Paralititan*, it obviously falls short of explaining sauropod gigantism in general, with their need to occupy huge land masses (Burness *et al.*, 2001).

Links between gigantism and particular food resources may be suggested by the rich worldwide and temporally extensive record of sauropod footprints from tidal flat sediments (Lockley & Meyer, 2000). Particularly in Upper Jurassic and Lower Cretaceous peritidal carbonate rocks, so-called megatracksites are preserved that cover thousands of square kilometers and show that sauropods lived in or migrated into the tidal flats several hundred kilometres from the nearest coast. Modern sedimentary environments of this kind are generally devoid of vertebrate life, and it remains unclear what the food base for the sauropods would have been. One possiblity are the Cheirolepidiaceae, an extinct conifer family, some members of which were succulent halophytes (Gomez *et al.*, 2002). However, based on the carbon isotope composition of sauropod bones and teeth, intensive feeding on marine food resources, such as algae or other marine plants, can be excluded (Tütken, in press). Nothing is known about the isotopic signature of Cheirolepidiaceae, though.

## VI. MORE RESOURCES AVAILABLE THROUGH EVOLUTIONARY INNOVATION

### (1) Long neck

We now want to explore evolutionary innovations that may have made more resources available to the individual, leading to gigantism in sauropods (Figs 6, 8). The most important of these is the hallmark of sauropod anatomy, the long neck. Potential selective advantages conferred by the long neck can be framed as two hypotheses. The first hypothesis is that the long neck allowed adult sauropods to exploit food resources beyond the reach of other large herbivores or smaller individuals of the same sauropod species, e.g. plant matter high above the ground. The second hypothesis is that a long neck and the resultant large feeding envelope would have conveyed a considerable energy savings in feeding as opposed to moving the whole body while feeding.

#### (a) First hypothesis: extension of reach

A very long neck obviously allows access to food at great heights, i.e. in the crowns of trees, if such a neck can be raised sufficiently high. Alternatively, sauropods that were unable to raise their neck could have accessed the additional resources by rearing up on their hindlimbs (Dodson, 1990; Paul, 1998; Mallison, in press *a*). In addition, during periods of food shortage, the ability to reach resources that could not be exploited by other animals would have carried a high selective advantage (Sander *et al.*, 2009). A similar selective advantage could have existed in low-browsing sauropods as well, e.g. if low-growing swamp plants such as stands of horsetails were not accessible or only difficult to reach without a long neck (Sander *et al.*, 2009). The hypothesis of the long neck greatly increasing the food resources available to a very large terrestrial herbivore thus seems to be well supported (Preuschoft *et al.*, in press).

#### (b) Second hypothesis: large feeding envelope versus acceleration of whole body

The energetic advantage of feeding with a long neck over covering the same feeding volume by walking depends on several factors, especially the distribution of food, the size of the animal, and the mechanical construction of the neck (Preuschoft *et al.*, in press). With respect to the costs associated with travel during foraging, it is mainly the acceleration of the huge body that is energy-expensive, not so much the travel itself. Shipley *et al.* (1996, p. 242) modeled the influence of locomotion on foraging behaviour in modern herbivores and state that an “animal may choose to exploit many bites at one ‘feeding station’ before moving on” over foraging continuously because of the considerable cost incurred from acceleration and deceleration. This means that an adaptation that enables more bites per feeding station would be advantageous for any animal—regardless of its body size. Obviously, a long neck is such an adaptation (Preuschoft *et al.*, in press). The interesting question resulting from this insight is why such long necks are not more common in herbivorous animals. If chewing, i.e. a dental battery, is in the adaptive repertoire of a lineage, then long necks will not be an option due to the disproportional increase in size and mass of the ingestive apparatus with increasing body size. This positive head allometry is apparent both in mammals, e.g. in horses (MacFadden, 1994), and in ornithischian dinosaurs (Long & McNamara, 1997*a*, *b*). Herbivorous birds, e.g. geese, lack a dental battery and have long necks, as do basal sauropodomorph dinosaurs from which sauropods must have arisen.

Applied to sauropods, moving the neck during feeding was not very energy-expensive. The neck could have been kept at the different inclinations by strong ligaments (e.g. Alexander, 1985, 1989; Dzemski & Christian, 2007; Schwarz *et al.*, 2007*a*) and muscles with slow fibres, so that little energy was required to keep the neck in a certain posture. During feeding, slow sideways movements of the neck probably were predominant and would have served to cover systematically the feeding envelope. Quicker and forceful changes of the position of the head could have been accomplished by flexion in the cranial neck section, so that only a small fraction of the body mass was involved in activities with high energy expenses. These assumptions fit the mechanical analyses of Christian & Dzemski (2007, in press) and Dzemski & Christian (2007). However, feeding on large trees requires great flexibility in the neck, so that the head can be moved in a maze of branches. Sauropods with very long cervical ribs (e.g. *Mamenchisaurus*) were thus probably not capable of accessing a larger three-dimensional volume by rearing up, simply because their necks would be too immobile (Mallison, in press *a*). By contrast, Diplodocidae had very mobile necks that potentially allowed treetop navigation.

If we assume that feeding on low vegetation (e.g. *Equisetum*) was important for sauropods, rather than on food distributed at different heights, then we would expect a positive allometry of neck length in order to compensate for the increasing distance between the origin of the neck and the ground in larger animals. Neck allometry is indeed positive interspecifically in sauropodomorphs (Parrish, 2006) and intraspecifically (Britt & Naylor, 1994; Ikejiri *et al.*, 2005; Schwarz *et al.*, 2007*b*). We conclude that this second hypothesis is supported as well. The selective advantage of the long neck of sauropods was the ability to exploit food sources that could not be reached by other herbivores or by smaller individuals of the same species and a considerably energy savings in feeding as opposed to moving the whole body while feeding.

### (2) Feeding

There are three ways for sauropods to have obtained more resources through feeding: by consuming more fodder, by feeding more selectively on the most nutritious plant parts such as seeds and young shoots, or by consuming plants with a higher energy content than today's vegetation (a boundary conditon hypothesis, discussed in Section V.2*a*). Consumption of greater quantities of food would appear to be an unlikely option because the head of sauropods is not disproportionally larger than expected for herbivores of their size (Paul, 1998; Christiansen, 1999). The previously hypothesized avian-style gastric mill in sauropods (e.g. Christiansen, 1996) would also have limited the rate of food intake (Sander & Clauss, 2008), but Wings & Sander (2007) showed that sauropods probably did not have a gastric mill. As time required for mastication was no constraint for sauropods, plant anatomy—how much material can be harvested in one bite—might have become a crucial determinant of intake, and might thus have determined sauropod foraging decisions (Hummel & Clauss, in press). However, it should be noted that the lack of a modern analogue—an endothermic herbivore without adaptations to mastication or to grinding in a gizzard, both of which are intake-limiting factors—leaves us speculating whether sauropods—and other herbivorous dinosaurs with similar characteristics—could have achieved higher food intake rates than extrapolated from mammalian herbivores.

Also unlikely is the hypothesis that sauropods were highly selective feeders on the most nutritious plant parts, i.e. seeds and young shoots. Known as the Jarman-Bell Principle, herbivores become less selective with increasing body mass, and all modern megaherbivores are bulk feeders (Owen-Smith, 1988; Cameron & du Toit, 2007). On the one hand, this is an effect of the increasing disparity in plant and oral anatomy with increasing body size. As herbivores become larger, they lose the ability to select only the most nutritious parts of plants which tend to be small (buds, seeds *etc*.). Also, due to their larger (more clumsy) mouth parts, they have to ingest larger chunks of food. On the other hand, because lower quality food is much more abundant than higher quality food, large herbivores cannot afford to search for the more dispersed high-quality food items due to their very high absolute food intake requirement (Demment & Van Soest, 1985). Note however, that this does not mean that large herbivores cannot subsist on high-quality food if it is abundant. Renecker & Hudson (1992) provide an excellent review, using the moose (*Alces alces*) as an example of a large herbivore that, if high-quality food is abundant, can subsist on it.

### (3) Greater digestive efficiency

The hypothesis of particularly efficient digestion is difficult to test in extinct animals. Nevertheless, one might be tempted to pursue such speculations based on the enormous body mass of sauropod dinosaurs, given the widespread notion that digestive efficiency increases with increasing body mass in herbivores (Demment & van Soest, 1985). This concept relies on the belief that ingesta retention time—the time that the ingested food stays in the gut and hence can be digested—increases with body mass (Illius & Gordon, 1992). This view has also been adopted for dinosaurs (Farlow, 1987; Midgley *et al.*, 2002). In a literature review of available data for mammalian herbivores, however, the generality of this relationship has been rejected (Clauss *et al.*, 2007, 2008*b*) because, among large mammalian herbivores, ingesta retention time does not consistently increase with body mass.

The concept of increasing digestive efficiency with increasing body mass has other limitations, too: with increasing body mass, the fineness that forage can be masticated into decreases in mammals—in other words, larger herbivores ingest larger particles (Fritz *et al.* 2009), which are more difficult to digest. Also, the absorptive surface of the gut increases with body mass as M^0.75^ whereas gut capacity increases as M^1.0^ (Clauss & Hummel, 2005; Clauss *et al.*, 2007), leading to less absorptive area per unit of ingesta. Among ruminants, energetic losses due to methane production increase with body mass (Clauss & Hummel, 2005). Data compilations from the literature have so far not supported the conclusion that larger animals achieve higher digestion coefficients (Pérez-Barbería *et al.*, 2004; Clauss & Hummel, 2005; Clauss *et al.*, 2009). Rather, among mammalian herbivores, different solutions for the interplay between intake, ingesta retention time, chewing efficiency, and digestive efficiency have been reached by different species and taxonomic groups (Clauss *et al.*, 2009). In particular, subtle differences in metabolic rate need to be taken into account when explaining this variation (e.g. Schwarm *et al.*, 2006).

One important constraint was recognized by Demment & Van Soest (1985), namely that digestive efficiency cannot be optimized endlessly, but is limited by the quality of the forage itself. To put it simply, digestive efficiency can only approach 100%, but it cannot increase further. Thus, increased ingesta retention in larger-bodied animals may be beneficial if offsetting the negative effect of decreased particle size reduction (as expected in non-chewing sauropods), but further increases in retention time probably will have a negative effect (Clauss *et al.*, 2003*a*). The optimal ingesta retention time for a herbivore of a given efficiency in particle size reduction will always be limited by the maximum digestibility of the forage it feeds on.

Large sauropods probably digested their forage with a similar efficiency to many extant mammalian herbivores. Other strategies observed in mammals, such as very inefficient fibre digestion offset by very high forage intake rates (in giant pandas, *Ailuropoda melanoleuca*), appear unlikely for sauropods—or they would have been particularly habitat-destructive and would not plot above the regression line in Burness *et al.* (2001) (Fig. 3). Such a presumed ‘efficient digestion’ in sauropods would entail long ingesta retention times, which may have allowed the exploitation of resources that were not attractive to smaller animals (Franz *et al.*, 2009). A case in point is the slow energy release pattern observed for *Araucaria* as discussed above (Hummel *et al.*, 2008) (Section V.2*a*).

### (4) Avian-style respiratory system

Increased oxygen uptake by the lungs of a sauropod dinosaur compared to a mammal of the same size would potentially allow gigantism. The tissues would be supplied with more oxygen, allowing a higher growth rate and a faster cell metabolism, making the organism work more efficiently on the same resources. Such a highly effective lung is seen in modern birds which extract about twice as much oxygen per unit air volume as mammals do. Two features of the avian lung make this possible: the air sac system which continuously provides fresh air to the parenchymal tissue of the lung and the crosscurrent gas exchange in this tissue (Perry, 1983, 1989, 1992).

Perry (1983, 1989, 1992) originally hypothesized that some saurischian dinosaurs had avian-style lungs. In recent years this has been generally accepted for the theropod lineage (Perry, 2001; O’Connor & Claessens, 2005; O’Connor, 2009) based on careful anatomical observations of pneumaticity in the skeleton and on phylogenetic arguments with birds as surviving theropod dinosaurs (O’Connor & Claessens, 2005; O’Connor, 2009). In the sauropodomorph lineage of Saurischia, a consensus is also emerging that the great extent and specific pattern of pneumatization of the precaudal part of the axial skeleton is evidence for an air sac system (see above, Perry, 2001; Wedel, 2003*a*, *b*, 2005, 52009; O’Connor & Claessens, 2005; Schwarz & Fritsch, 2006; Schwarz *et al.*, 2007*a*; O’Connor, 2009). Thus, a hypothetical avian-style lung in sauropods is compatible with the evidence (Perry & Reuter, 1999).

Applying this insight to a mathematical model leads to the recognition that an avian-style lung in sauropod dinosaurs indeed would have greatly increased respiratory efficiency (Perry *et al.*, 2009, in press). In addition, a highly efficient avian-style lung would mean that the volume of the gas exchange part (exclusive of the air sacs) would be small, and gravitational influence on the respiratory system even of a large sauropod would not be constraining (Perry & Reuter, 1999). This is because of the small vertical extent of an avian-style lung which obviates the need to raise the blood within the lung against gravity. This presents a problem in very large mammals such as elephants and led to the evolution of special support structures (Perry *et al.*, 2009, in press).

## VII. FEWER RESOURCES USED

In order to understand fully the importance of reduced resource use for sauropod gigantism, the energy budget of a living sauropod dinosaur would have to be reconstructed, as attempted by Weaver (1983). However, the different pathways of energy uptake and energy expenditure are now known to be much more complex then envisaged by Weaver (1983). While highly desirable, quantification remains the subject of future research and qualitative considerations must suffice here.

The energy budget of an animal is divided into consumption of energy for growth, maintenance, thermoregulation, support, locomotion, respiration, feeding, and reproduction. In the following, we evaluate the possible ways that sauropod dinosaurs could have conserved energy through evolutionary innovations and scaling effects and thus made better use of the resources available to them than similar-sized mammalian megaherbivores and ornithischian dinosaurs would have been able to.

### (1) Reduction in body density

Body mass (and its distribution) fundamentally influences the static and kinetic energy requirements of an organism, and the reduction of body mass relative to linear dimensions, i.e. reduction of specific body density, will convey a major energetic advantage (and thus selective advantage, Currey & Alexander, 1985; Wedel, 2005, 2009). This leads to the hypothesis that reduction in specific body density made the gigantism of sauropod dinosaurs possible. Bone, being the densest tissue in the skeleton and also the one that is most accessible to palaeontologists, is the obvious focus for testing this hypothesis. One way to reduce skeletal mass is to evolve particular light-weight constructions and the other is to evolve materials of superior strength.

#### (a) Superior skeletal materials

This hypothesis can be framed as follows: sauropod bone tissue may differ at one or more hierarchical levels in its physical properties from that of other tetrapod bone, making it significantly stronger mechanically. This, in turn, would allow more slender or thinner bones and result in a lower specific density of the animal.

The hypothesis was tested using the approach of materials science, where materials are investigated for their structure at all hierarchical levels, from shape to nanostructure. Pyzalla *et al.* (2006*a*, *b*) and Dumont *et al.* (2009, in press) have recently taken this materials science approach to sauropod bone, focusing on primary fibrolamellar bone, the dominant tissue type in the cortex of sauropod long bones (Klein & Sander, 2008; Sander *et al.*, in press *b*). They compared sauropod primary fibrolamellar bone to fibrolamellar and Haversian bone in large mammals using X-ray diffraction, proton-induced X-ray emission (PIXE) spectroscopy, and other methods for eludicating hierarchical structure. These methods indicate that sauropod bone retains its original crystallite orientation and that its microstructure at the different hierarchical levels appears to be the same as that of modern bone. Current evidence thus rejects the hypothesis that sauropod dinosaur bone was an unusually high-strength material.

#### (b) Light-weight construction

Light-weight constructions are well known in nature and have frequently evolved, their study being a focus of the field of biomechanics. With the discovery of the cavernous nature of the cervical and dorsal vertebral column in all but the most basal sauropods, it has been argued that the sauropod vertebral column was such a light-weight construction. However, only through the more detailed study of vertebral pneumaticity enabled by computed tomography (Wedel, 2000*a*, 2003*b*, 2005, 2009; Schwarz & Fritsch, 2006; Schwarz *et al.*, 2007*a*) and histology (Woodward & Lehman, 2009), has the quantification of weight reduction in the vertebral column been possible (see section I.3). The extensive air sac system of sauropods with diverticula invading most of the presacral vertebral column and the ribs resulted in a specific body density of 0.8 kg L^−1^, with certain parts such as the neck having a value of 0.6 kg L^−1^ only (Henderson, 2004; Wedel, 2005; Schwarz & Fritsch, 2006). This is also expressed as a body mass reduction by 8–10% in volume-based estimates (Wedel, 2005). The hypothesis that the light-weight construction of the axial skeleton of sauropods contributed to their gigantism thus is supported. Interestingly, the largest land mammal, *Paraceratherium*, had pleurocoel-like openings in the presacral vertebrae, but it is not known what these were filled with and whether they contributed to lightening the skeleton.

### (2) Reduced cost of locomotion

Locomotor activity of an animal represents one of the most important components of its energy budget (Biewener, 2003), leading to the hypothesis that improved scaling of the cost of locomotion would have allowed sauropod gigantism by slowing down the increase in overall energy uptake with evolutionarily increasing body size. In addition to scaling effects, design of the locomotory apparatus needs to be taken into consideration. While the cost of transport will decrease per unit of body mass (Langman *et al.*, 1995; Alexander, 2006), this relationship has not been studied quantitatively in sauropods. As graviportal animals with long legs, the general sauropod locomotory design resembles that of graviportal mammals, leaving scaling effects as the greatest potential energy savings. Since much of locomotion in sauropods may have been linked to feeding (protection from predators not having been an issue), locomotion and the the long neck should be considered together (see Section VI.1).

### (3) Reduced cost of respiration

In addition to providing the organism with more oxygen (see Section VI.4), better oxygen uptake through a bird-like respiratory system would translate into energy conservation because breathing involves muscular work and thus energy consumption. However, the contribution to the energy budget of a living sauropod would have been relatively small because the muscles involved in breathing would have been only a small fraction of the muscle mass of the animal. Furthermore, the presence of large air sacs would result in a low-frequency breathing pattern. Birds have a greater tidal resting volume and lower breathing frequency than mammals of the same body mass. Since the work of breathing and its energetic cost is directly proportional to breathing frequency and inversely proportional to the compliance of the respiratory system, an avian-like lung-air-sac system in a sauropod would be extremely energy-efficient to operate. The result in the case of a bradymetabolic homoiothermic giant sauropod would be an extremely low energetic cost of breathing per unit time compared with extant mammals and birds (Perry *et al.*, 2009). In a tachymetabolic homoiotherm, the energetic cost of breathing per unit oxygen acquired would be absolutely higher, because of the higher metabolic rate, but relatively still much lower than in a mammal-like lung.

### (4) Lower basal metabolic rate and gigantothermy

Body heat, whether generated metabolically (as in endotherms) or being taken up from the environment (as in ectotherms), is central to the energy budget of an animal. If sauropods had a lower BMR than mammalian herbivores in extant ecosystems, it would have allowed the former to evolve a larger body size (see also Burness *et al.*, 2001; McNab, 2009). A lower metabolic rate would not have triggered gigantism in itself, but rather permitted other evolutionary factors to push body size to extremes (McNab, 2009). However, the hypothesis of a lower BMR is contradicted by ample evidence (as reviewed in Section II.11) for sauropods having been tachymetabolic endotherms, at least during the phase of active growth lasting for most of their life history. Specifically, as noted by McNab (2009), a hypothetical sauropod dinosaur with a basal metabolic rate of a varanid cannot be reconciled with the high growth rates inferred for sauropods. Although energetic scaling effects and gigantothermy may have represented a contributing factor to gigantism, saving resources by having been bradymetabolic throughout ontogeny as the explanation for sauropod gigantism thus must be rejected (*contra*McNab, 2009). This hypothesis is open to further testing by a comprehensive model of the energy budget of a living sauropod, including the potentially drastic reduction in BMR during ontogeny.

### (5) Reduced cost of reproduction

Another important part of the energy budget of an animal is taken up by reproduction, albeit with a more episodic energy expenditure. It thus could be hypothesized that sauropod dinosaurs employed a more energy-efficient mode of reproduction than other dinosaurs and large mammals. This would have to be sought in the ovipary of sauropods and possibly in the relatively minute eggs they produced (Case, 1978; Weishampel & Horner, 1994; Sander *et al.*, 2008). Available evidence consists of the eggs and clutches of the Late Cretaceous fossil egg taxon *Megaloolithus* which occurs around the world, particularly in Europe, India, and Argentina. All except the Argentinian eggs were buried in the substratum or under plant matter and are found in small clutches of less than ten eggs (Sander *et al.*, 2008). If only one clutch was produced by the female each season, this would amount to a very small parental reproductive investment, especially since any form of parental care appears unlikely (Mueller-Töwe *et al.*, 2002; Sander *et al.*, 2008). Charnov, Warne & Moses (2007) showed that the average lifetime reproductive effort ]LRE, defined as (litters or clutches per year) x (litter or clutch size) x (average adult life span) x (offspring mass at independence/adult mass at first reproduction[, where the latter quotient measures the degree of parental care on average does not differ between mammals and lizards, and thus LRE is approximately similar in these major vertebrate groups. Based on the comparison with modern species, we reject the hypothesis of more energy-efficient reproduction in sauropods. Nevertheless reproduction and life history are important in understanding sauropod gigantism.

## VIII. FASTER POPULATION RECOVERY AND FASTER INDIVIDUAL GROWTH

### (1) Ovipary and gigantism

Their oviparous, more *r*-selected mode of reproduction (Section II.11) may have been a major contributing factor to sauropod gigantism. This hypothesis was first advanced by Janis & Carrano (1992) for dinosaurs in general (see also Farlow, 1993) and later applied specifically to sauropods by Paul (1994, 1997b). Based on a large dataset of number of offspring *versus* body mass for mammals and birds, Janis & Carrano (1992) noticed that the number of offspring decreased significantly with body size for mammals while this was not the case in birds, where it remained constant. They then hypothesized that because all dinosaurs were oviparous, the same relationship might have applied, and that ovipary enabled dinosaurs to achieve a greater body mass than mammals because the greater reproductive output of large dinosaurs led to a lower risk of chance extinction than for similar-sized mammals.

Further support for their hypothesis comes from other macroecological analyses. Positive correlation of clutch size and body size is documented for turtle species (Frazer, 1986), for snakes (Ford & Seigel, 1989) and for reptiles in general (Blueweiss *et al.*, 1978; King, 2000). No relationship was found for galliform birds (Kolm *et al.*, 2007), as was stated by Janis & Carrano (1992). The difference between reptiles and birds may be explained by their internal thermal conditions (Shine, 2005). The data for modern reptiles also show that increasing clutch size with increasing body size alone is insufficient to explain gigantism (otherwise there would be widespread gigantism in modern reptiles).

The argument of Janis & Carrano (1992) is linked to the selective disadvantages of large body size (Blankenhorn, 2000; Hone & Benton, 2005), specifically that large body size increases the risk of extinction of a species. This is because long generation times decelerate evolutionary adaptation processes, and the increased demand for resources of the individual will lead to lower population densities. The increased risk of demographic population extinction caused by low densities is also at the centre of the hypothesis of Burness *et al.* (2001) that maximum body size is limited by land mass size. The hypothesis of Janis & Carrano (1992) lends itself to a modelling test using evidence for sauropod reproduction and population turnover that has become available since their study (e.g. Erickson, 2005; Sander *et al.*, 2008) and combining it with the body-size-area relationship of Burness *et al.* (2001). However, in a qualitative fashion we already identify the production of many small offspring allowing fast population recovery as an important factor contributing to sauropod gigantism.

### (2) Survivorship, high growth rate, and high BMR

Adult sauropods presumably were almost immune from predation because their body mass was an order of magnitude greater than that of the largest predators. Their sheer volume made it difficult for an attacker to place an effective bite rather than scratch the skin (Preuschoft *et al.*, in press). With sauropod hatchlings being so small, there must have been strong selection pressure for high juvenile growth rates because they would have shortened the time during which the young sauropods were endangered by predators. Selection for high growth rates would have been particularly strong without parental care. In more general terms, a high growth rate fueled by a high BMR are prerequisites to giant body size because tetrapods with a low BMR grow too slowly to benefit from the selective advantages of large body size. A high, i.e. mammalian or bird BMR thus emerges as a prerequisite for gigantism, while a reptilian BMR limits body size to around 1 t under current environmental conditions (Makarieva *et al.*, 2005; Head *et al.*, 2009).

## IX. HISTORICAL CONTINGENCY

In addition to hypotheses addressing sauropod gigantism from a bauplan limitation or a resource perspective, there have been repeated attempts in the literature to explain this phenomenon as the result of a historical evolutionary process. However, these hypotheses suffer the general problem of historical hypotheses in that they may explain how a certain species or group outcompeted another one but not why sauropods were ‘uniquely free of the size constraints evident in other groups' (Carrano, 2006, p. 24).

### (1) Decreased oxygen content of atmosphere

Ward (2006) and Berner *et al.* (2007) suggested that the evolutionary success of the Saurischia in the Late Triassic, replacing rhynchosaurs as the major herbivores and therapsids and crurotarsan archosaurs as the major carnivores (Benton, 1990), was made possible by the avian-style respiratory system of the early saurischians. The Late Triassic was the time of the lowest atmospheric oxygen levels of the entire Phanerozoic, and the ability of taking up twice as much oxygen than other tetrapods would have been of great selective adavantage. This hypothesis is in accordance with several observations, e.g. both sauropods and theropods increased in body size very rapidly compared to ornithischian dinosaurs, and saurischian dinosaurs dominated the Jurassic faunas. However, as noted above, there is no positive evidence that basal sauropodomorph dinosaurs (prosauropods) had an air sac system and hence bird-like lungs (Wedel, 2007), although their presence might, of course, be reconstructed on phylogenetic grounds.

### (2) Poor food quality

Midgley *et al.* (2002) proposed that the low food quality of the pre-angiosperm vegetation would have driven the evolutionary increase of sauropod body size. Their hypothesis is based on the observation that in living mammals large body size correlates with low food quality. Implicitly they argue that the evolution of animals larger than the largest mammals was driven by the need for prolonged retention times of the low-quality fodder in ever larger guts which led to ever longer retention times. However, this hypothesis has flaws. First, the laboratory experiments of Hummel *et al.* (2008) have shown that many pre-angiosperm plants are no less nutritious than angiosperms. Second, sauropod dinosaurs continued to thrive in a world populated by angiosperms, i.e. during the Late Cretaceous. In addition, there is no evolutionary response to the mid-Cretaceous floral turnover in terms of a size decrease in sauropods (Barrett & Upchurch, 2005). Finally, the premise of Midgley *et al.* (2002) that retention time increases with body size may not be well supported (Clauss *et al.*, 2007; see also Section VI.3). We thus reject the hypothesis of Midgley *et al.* (2002).

## X. DISCUSSION

**Fig. 9:**
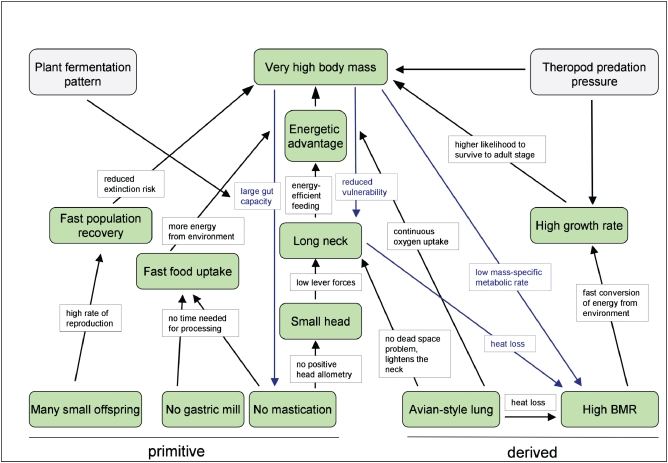
Flow chart of the evolutionary cascade leading to sauropod gigantism. The green boxes contain the biological properties of sauropods, and the black arrows indicate primary evolutionary causation. Theropod predation pressure is depicted as a representative selection factor for body size increase. In addition to primary evolutionary causation, sauropod gigantism was also driven by evolutionary feedback loops (blue arrows). The blue boxes indicate the selective advantage in the feedback loop. The boxes on the black arrows show the selective advantages conferred on sauropods by the biological properties. BMR, basal metabolic rate.

We are now able to identify those hypotheses that may explain gigantism and those that probably do not, based on a current (2010) understanding. This leads to the recognition that the gigantism of sauropod dinosaurs was made possibly by a unique combination of two retained plesiomorphies and three key evolutionary innovations (Fig. 9).

Probably the most conspicuous features of the sauropod bauplan, the very long neck, was the first key innovation in the evolution of gigantism. Its importance is supported by observations that the group with relatively and absolutely shortest necks, the Dicraeosauridae and Rebbachisauridae were significantly smaller than all other sauropod groups (Upchurch *et al.*, 2004; Sereno *et al.*, 2007), i.e. that neck length scales with positive interspecific allometry (Parrish, 2006). The long neck allowed exploitation of food inaccessible to smaller herbivores and a much larger feeding envelope than in a short-necked animal and thus significantly decreased the energetic cost of feeding (Stevens & Parrish, 1999; Preuschoft *et al.*, in press; Seymour, 2009*a*). It also must have been advantageous in that it greatly increased body surface area and thus the heat loss capacity of an exercising sauropod. The evolution of a long neck was biomechanically possible in sauropodomorphs because the head was small, not serving in mastication of the food, but only for gathering it. Non-mastication is the first of the plesiomorphic conditions retained in sauropods.

Mammals, on the other hand, were prevented from evolving long necks in large forms by their extensive mastication which necessitates a relatively large head to accommodate the dentition, a very strong masticatory musculature and very strong (=heavy) bony elements to sustain the resulting stresses, particularly as body size increases. As in mammals, extensive mastication of plant food in the major ornithischian dinosaur lineages Ornithopoda and Ceratopsia may have placed a constraint on their body size. Other, less obvious constraints originating from mastication were discovered through the fermentation experiments of Hummel *et al.* (2008). In these experiments, *Equisetum* had the highest energy content of any of the non-angiosperm plants. While disadvantageous to mammals because of their abrasiveness on chewing teeth, sauropods could extensively have relied on this resource because of their lack of mastication. This view is supported by recent work on the scaling and interplay of gut contents, food retention time, food intake rate, and degree of particle reduction that shows that sauropods could have compensated for large ingesta particle size with long retention times (Clauss *et al.*, 2009; Franz *et al.*, 2009).

The other major factor allowing the evolution of the long sauropod neck was their hypothesized avian-style respiratory system, which positively affected neck length in two ways: by allowing extremely light construction (Wedel, 2003*b*, 2005) and by solving the problem of tracheal dead space (Wedel, 2003*b*, Perry *et al.*, 2009, in press). The light-weight construction of the neck resulted from the extensive pneumatization of the neck vertebrae originating from the invasion of the axial skeleton by diverticula of cervical air sacs. Only with the storage capacity provided by the air sacs, could the problem of tracheal dead space facing long-necked mammals such as giraffes (Perry, 1983, 1992; Daniels & Pratt, 1992; Calder, 1996; Hengst *et al.*, 1996; Paladino *et al.*, 1997; Paul, 1998) be overcome.

Beyond facilitating the evolution of the long neck, the hypothesized bird-like respiratory apparatus offers additional advantages, emerging as the second key evolutionary innovation. These advantages include (*a*) pneumatization originating from air sacs greatly lightened the dorsal axial skeleton of the trunk without compromising its strength (Wedel, 2005). (*b*) The continuous-flow, cross-current lung would have increased oxygen uptake twofold per unit air breathed compared to the ventilated pool model of the mammalian lung (Fedde, 1990). This would have decreased the energetic cost of breathing while at the same time supplying the tissues with adequate oxgen. (c) The large internal surface of the trachea and air sacs in contact with the viscera and the neck would have provided ample possibility for excess heat loss which then was removed by exhalation from the body. An effective internal cooling mechanism presumably was crucial for sauropods during the phase of active growth when they had a high basal metabolic rate (Fig. 9C). We note that a respiratory system analogous to that of birds was recently hypothesized to have permitted gigantism in flying reptiles, the pterosaurs (Claessens, O’Connor & Unwin, 2009)

Ovipary is the second plesiomorphy retained in sauropods that permitted gigantism because it led to higher population recovery rates in these dinosaurs than in megaherbivore mammals (Janis & Carrano, 1992). As noted by Farlow, Dodson & Chinsamy (1995), the hypothesis of Janis & Carrano (1992) would predict gigantism in Tertiary birds in the form of multi-tonne ground birds. Since such animals did not evolve, other constraints may have been effective, such as the obligatory bipedalism of birds or competition from mammals.

From arguments rooted in evolutionary ecology, the high metabolic rate of sauropods is identified as the third key evolutionary innovation permitting gigantism because it fueled the high growth rate required by young sauropods to survive to sexual maturity (Dunham *et al.*, 1989). The high growth rate also increased population recovery rate because the numerous sauropod offspring must have grown quickly to reach sexual maturity. The low growth rate of ectothermic reptiles thus provides one explanation why lineages such as turtles and crocodiles were prevented from evolving to dinosaurian body size despite their positive scaling of offspring number with body size (Griebeler & Werner, in press).

Scaling effects (Alexander, 1989, 1998, 2006) will also have played an important role in sauropod gigantism, in particular with regard to locomotory efficiency and thermometabolism as detailed above, but may have been insufficient to release other constraints on body size by themselves. In addition, scaling effects would be insufficient to explain sauropod gigantism, since they would apply to other taxonomic groups as well.

This review rejects a number hypotheses about sauropod gigantism: there is no evidence for a higher atmospheric oxygen level during the Mesozoic than today. A higher level is not necessary for the sauropod body plan to function (*contra*Hengst *et al.*, 1996), assuming that sauropods possessed a bird-like respiratory apparatus. Higher ambient temperatures are also unlikely to have contributed to sauropod gigantism because there is no evidence that they do in modern endothermic tetrapods. Higher plant productivity caused by increased levels of atmospheric CO_2_ was at least partially offset by the decreased nutritious value of the plant matter. Finally, on the biotic side, there is no indication that sauropod bone tissue had mechanical properties superior to the fibrolamellar and secondary bone tissue of large mammals and that sauropods invested less energy into reproduction than other animals.

A major problem with virtually all hypotheses invoking different boundary conditions to explain gigantism is that their variation through Mesozoic time does not correlate with body size evolution in Sauropoda, nor with their diversification (Upchurch & Barrett, 2005). In particular, sauropod body size evolution neither tracks atmospheric oxygen levels, nor atmospheric CO_2_ levels, nor global temperature curves (Fig. 8). The only physical parameter that seems to be reflected in sauropod body size evolution is land mass size. This may be the explanation for the observation by Upchurch & Barrett (2005) that there appears to be a correlation between sauropod diversity and sea level. These authors, did not, however, test for a correlation between land mass size and sauropod diversity.

The goal of future work must be a model of the energy budget of a living sauropod and its comparison with that of large mammals. These data can then be combined with information on land mass size (Burness *et al.*, 2001) and carrying capacity to detect a possible coupling of land area and body mass in sauropod dinosaurs. We are not yet able to quantify the relative contributions of the three factors *more resources available*, *fewer resources used*, and *reproduction mode* (Fig. 7) to the solution to the land area *versus* body size enigma because in addition to data about the enery budget of a living sauropod, this will require understanding the energy flow in a sauropod ecosystem.

## XI. CONCLUSIONS

1. Sauropod dinosaurs as the largest terrestrial animals ever represent a challenge to evolutionary biologists trying to understand body size evolution.
2. The study of the upper limit of body size must address extrinsic as well as intrinsic factors, and it must be determined whether this limit is set by the bauplan of the organisms or by physical and ecological constraints imposed by the environment. Among several possible approaches, we chose the resource perspective because it has been shown that resource availability and maximal body size correlate closely (Burness *et al.*, 2001).
3. In the interplay of the biology of sauropod dinosaurs with their environment, a unique combination of plesiomorphic features (i.e., inherited from their ancestors) and evolutionary novelties emerge as the key for a more efficient use of resources by sauropods than by other terrestrial herbivore lineages. Plesiomorphic features of sauropods were many small offspring, the lack of mastication and the lack of a gastric mill. The evolutionary innovations were an avian-style respiratory system and a high basal metabolic rate.
4. We posit that the long neck of sauropods was central to the energy-efficient food uptake of sauropods because it permitted food uptake over a large volume with a stationary body.
5. In the Late Triassic and Early Jurassic (210–175 million years ago), the combination of biological properties listed above led to an evolutionary cascade in the sauropodomorph lineage characterized by selection for ever larger body size, mainly driven by predation pressure from theropod dinosaurs.
6. From the Middle Jurassic onward, sauropod dinosaurs dominated global terrestrial ecosystems only to succumb to the catastrophic environmental change at the end of the Cretaceous 65 million years ago.
